# Brain Delivery of Multifunctional Dendrimer Protein Bioconjugates

**DOI:** 10.1002/advs.201700897

**Published:** 2018-02-23

**Authors:** Pierpaolo Moscariello, David Y. W. Ng, Malin Jansen, Tanja Weil, Heiko J. Luhmann, Jana Hedrich

**Affiliations:** ^1^ Institute of Physiology University Medical Center of Johannes Gutenberg University Mainz Duesbergweg 6 D‐55128 Mainz Germany; ^2^ Max Planck Institute for Polymer Research Ackermannweg 10 55128 Mainz Germany

**Keywords:** blood‐brain barrier, drug delivery, PAMAM dendrimer, streptavidin

## Abstract

Neurological disorders are undoubtedly among the most alarming diseases humans might face. In treatment of neurological disorders, the blood‐brain barrier (BBB) is a challenging obstacle preventing drug penetration into the brain. Advances in dendrimer chemistry for central nervous system (CNS) treatments are presented here. A poly(amido)amine (PAMAM) dendrimer bioconjugate with a streptavidin adapter for the attachment of dendrons or any biotinylated drug is constructed. In vitro studies on porcine or murine models and in vivo mouse studies are performed and reveal the permeation of dendronized streptavidin (DSA) into the CNS. The bioconjugate is taken up mainly by the caveolae pathway and transported across the BBB via transcytosis escaping from lysosomes. After transcytosis DSA are delivered to astrocytes and neurons. Furthermore, DSA offer high biocompatibility in vitro and in vivo. In summary, a new strategy for implementing therapeutic PAMAM function as well as drug delivery in neuropathology is presented here.

## 1 Introduction

Neurological diseases are a growing challenge in health care since, with prolonged aging, the number of patients will increase, with consequent high social impact due to severe morbidity and mortality.^1^ Although the scientific achievements are constantly providing possible therapeutic molecules,^2^ adequate treatments are still an unmet medical need, because systemically administered drugs are often ineffective due to a well‐known biological obstacle: the blood‐brain barrier (BBB). The BBB consists of brain capillary endothelial cells, pericytes, astrocytes, and neurons all referred to as the neurovascular unit (NVU).^3^ It represents a multicellular interface separating bloodstream and brain parenchyma, maintaining a constant homeostatic brain environment. After intravenous injection, only some lipophilic molecules penetrate to the brain parenchyma in therapeutically relevant concentrations, and 98% of neuroactive drugs cannot pass the BBB.^4^ Currently, therapeutics can be delivered to the central nervous system (CNS) via several ways. Either the drug can circumvent the BBB after systemic administration or it has to be applied by invasive methods involving a high risk of severe side effects.^5^ Possible approaches are, for example, opening of the tight junctions by osmotic disruption^6^ or ultrasound^7^ and direct intracerebral infusion or implantation.^8^ In rare cases, such as traumatic brain injury or cancer, the pathological mechanisms by themselves, affecting BBB integrity, might offer the possibility to access the CNS.^9^

Since virtually every neuron has its own connection to a microvessel,^10^ the approach to deliver drugs via the NVU interface is one of the most promising strategies to efficiently target the brain.^11^ In the past few years various nanoparticle (NP) types have been investigated to develop nanosystems meeting the needs of effective therapies for neurological diseases,^12^ also leading to a broader understanding of the mechanism of NP uptake in the brain.^13^ Among the many achievements, dendrimers showed high potential for a noninvasive therapy. Several examples of inherent dendrimeric therapeutic potential have been presented: (a) antiprion,^15^, ^16^ b) antitoxin,^17^ and c) antiamyloidogenic^18^ effects. Additionally, dendrimers represent a perfect platform for CNS drug conjugation. For several neuropathologies multiple suitable dendrimer–drug conjugates have been introduced to the scientific community, like for the treatment of (a) Parkinson's disease (dendrigraft poly‐l‐lysine‐PEG‐Angiopep),^19^ (b) hypoxia‐ and stroke‐mediated hypoxia (HIF‐1α siRNA/2G‐NN16 carbosilane dendrimer),^20^ (c) glioblastoma (G3‐succinamic acid dendrimer‐curcumin),^21^ and (d) HIV‐1 infection (2G‐(SNMe3I)11‐FITC cationic carbosilane).^22^ For instance, Kannan and co‐workers have published an impressive history in dendrimer applications improving the treatment of neuroinflammation. Observed uptake of poly(amido)amine (PAMAM) dendrimers in activated microglia and astrocytes in a model of cerebral palsy (CP) lead to the development of a successful dendrimer‐based N‐acetyl‐l‐cysteine (D‐NAC) therapy of CP.^23^ With neutral dendrimers a rapid 100‐fold greater brain uptake compared to free drug could be achieved.^24^ In a recent study the generation effect of hydroxyl PAMAM dendrimers in a canine model of induced brain injury could reveal much better pharmacokinetics and biodistribution of G6 dendrimers compared to G4 dendrimers.^25^ Furthermore, a successful antineuroinflammatory effect could be obtained for the treatment of the Rett syndrome, a pervasive developmental disorder. Although in this case D‐NAC significantly improved behavioral outcomes, it could not prevent the lethal outcome.^26^ The need of very careful investigations presenting new CNS therapeutic strategies was revealed by the case of poly(propyleneimine) glycodendrimers, which offer antiamyloidogenic effects but at the same time cause cognitive decline in WT mice.^18^ Despite the investigated and described dendrimer systems for CNS applications, dendrimers with high biocompatibility, new linker strategies, and possibilities to transport biologicals (e.g., therapeutic proteins) are eagerly awaited as highlighted in the review articles by Leiro et al. and Mignani et al.^14^, ^27^

**Figure 1:**
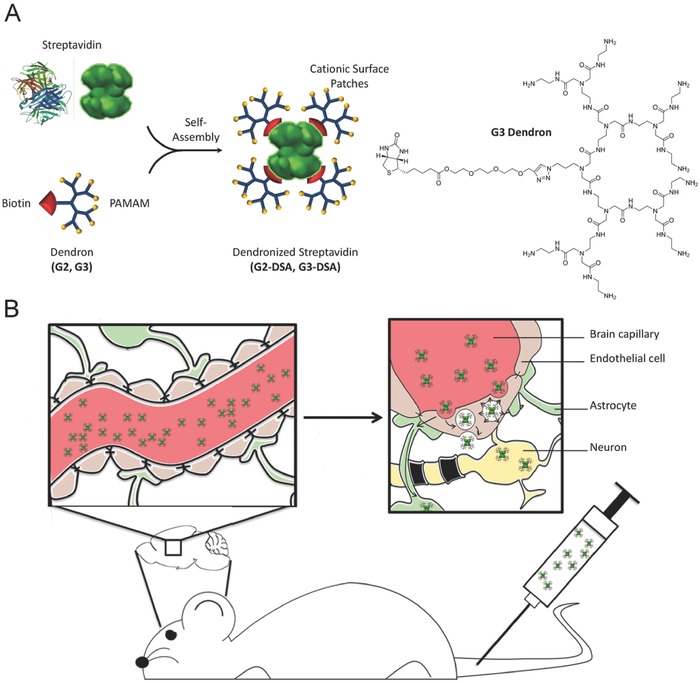
DSA synthesis and investigated mechanism of biological application. A) Scheme of dendronized streptavidin assembly with detailed G3 dendron structure. B) Schematic illustration of in vivo **s**tudy design for G3‐DSA transport from bloodstream to the brain.

Dendrimers are highly branched globular macromolecules built of branched monomers on a small core molecule. The advantages of using dendrimers are control over size, liquid solubility, and a multitude of functionalization possibilities.^28^ However, dendrimer synthesis is laborious and the balance between toxicity and biodegradability is highly dependent on the scaffold. Indeed, non‐biodegradable dendrimers are highly associated with in vivo toxicity. Therefore, the use of biodegradable dendrimers is strongly encouraged for biomedical applications.^29^ Since the core of a dendrimer is generally inconsequential toward targeting and recognition, substituting it with a protein core provides an elegant approach toward both synthesis and biocompatibility without compromising the monodispersed nature of both species. Specifically, the dendronized streptavidin (DSA) mimics endogenous proteins with a size of 5 nm and a patched surface using a streptavidin adapter coated by PAMAM dendrimers (DSA) (**Figure** **1**A). To our knowledge, the presented simple and elegant dendrimer system would allow for the first time to employ both inherent therapeutic functions of PAMAM dendrimers^16^, ^17^, ^30^, ^31^ and therapeutic effects of multiple drugs (including biologicals) in the same system attached to the streptavidin core by biotin‐click‐chemistry obtaining a new flexible synergistic nanoplatform.

Two generations of PAMAM dendrimers with a different number of positive charges were designed and their ability to cross the BBB and reach target cells in the CNS combined to a streptavidin adapter was assessed.

In our study, we have investigated the most relevant features of the bioconjugate and we could successfully address the following questions: (I) Are DSA taken up by endothelial cells and transported in an in vitro BBB model from luminal to abluminal? (II) Which are the underlying mechanisms of the transport? (III) Does the generation of PAMAM dendrimers influence uptake and transport efficiency? (IV) Do DSA affect vitality of NVU cells? (V) Are DSA able to cross the BBB and how are they biodistributed in vivo? (VI) Do they affect the BBB integrity in vivo?

We could successfully demonstrate that DSA were taken up by the endosomal pathway, transcytosed in vitro and in vivo across the BBB and taken up in target cells with higher efficiency for the third generation of DSA (Figure 1B). A high biocompatibility could be observed in impedance analysis (electric cell–substrate impedance sensing (ECIS), epithelial volt/ohm meter (EVOM), CellZscope), in viability assays in vitro, as well as in Evans blue (EB) permeability assays in vivo.

With this new generation nanosystem, we aim to overcome the BBB with a noninvasive and biocompatible compound, which employs transcytosis as an endogenous transport system in order to apply PAMAM dendrimer antitoxin, antiprion or antiviral function in CNS or even deliver biopharmaceuticals (macromolecules) to the brain with high efficiency.

## 2 Results and Discussion

### 2.1 DSA Are Taken Up by Brain Endothelial Cells In Vitro

**Figure 2:**
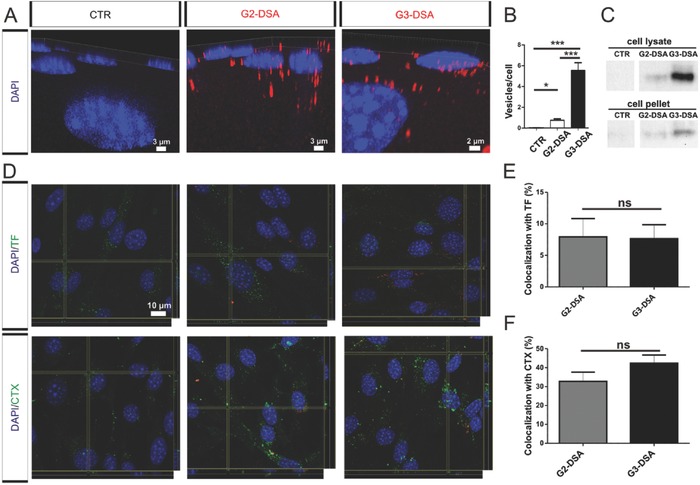
DSA uptake in bEnd.3 cells. A) Confocal 3D‐image of DSA (red) uptake. B) Quantification of DSA‐positive vesicles per cell. *n* = 28 ROIs from three cultures, one‐way ANOVA, ***p < 0.001, *p < 0.05. C) Streptavidin immunoblotting of cell lysate after DSA uptake. D) Confocal orthogonal view of DSA (red) couptake with TF or CTX (green) in bEnd.3 cells. E,F) Percentage of colocalization of DSA with FITC‐TF (E) or FITC‐CTX (F). *n* = 20 ROIs from three independent experiments, Mann‐Whitney U test, ns = not significant.

The tightly connected layer of brain endothelial cells represents the first obstacle to the entry of molecules into the CNS. Therefore, we investigated the ability of differently charged DSA (G2‐DSA and G3‐DSA) to be taken up by bEnd.3 cells in vitro. After 24 h incubation with DSA (rhodamine covalently labeled streptavidin), 3D reconstructions of confocal z‐stacks revealed the presence of NP‐containing‐vesicles in the cell cytosol of treated samples compared to control (CTR) untreated cells (**Figure** **2**A). Quantitative analysis showed significant uptake for both generations of DSA with a significantly higher number of NP‐containing‐vesicles for G3‐DSA than for G2‐DSA (CTR: 0 ± 0 vs G2‐DSA: 0.7 ± 0.7 vesicles per cell, CTR: 0 ± 0 vs G3 5.2 ± 0.43 vesicles per cell, G3‐DSA: 5.2 ± 0.43 vs G2‐DSA: 0.7 ± 0.7 vesicles per cell, *n* = 28 regions of interest from three cultures, one‐way ANOVA, *P* < 0.0001) (Figure 2B). Due to the protein core of DSA, fluoro‐cytochemistry experiments could be matched to a biochemical approach using western blot (WB) carried out on cell lysate from DSA‐treated cells. Detection of the NP core in an independent manner from the fluorescent signal excluded false positive data by free fluorescent‐dye. NP signal was detected in cell lysate as well as in cell pellet confirming the uptake of DSA in bEnd.3 cells (Figure 2C). Furthermore, a complete degradation of the protein core within 24 h could be excluded. These data indicate that differences in the structures and in the amount of positive charges of the dendrimers trigger the ability of DSA to penetrate brain endothelial cells with a more pronounced uptake for G3‐DSA than for G2‐DSA. In order to exclude a possible positive influence of rhodamine label on uptake efficiency, bEnd.3 cells have been treated for 24 h with Cy5‐labeled DSA. Our results revealed highly comparable cell uptake between Rhodamine‐ and Cy5‐ labeled DSA (data not shown).

The most common uptake mechanism in endothelial cells is the caveolae‐mediated uptake^32^ but it has been demonstrated that PAMAM dendrimers are taken up via a clathrin‐mediated pathway.^33^ To better understand the underlying uptake mechanism, we investigated the couptake of DSA with markers for both pathways. For that, FITC‐transferrin (TF) for clathrin‐mediated endocytosis and the subunit B of cholera toxin‐alexa‐fluor‐488 (CTX) for the caveolae‐mediated endocytosis were applied in combination with DSA for 24 h to follow the two pathways. Confocal microscopy showed colocalization of DSA with both investigated endocytosis markers (Figure 2D). Quantitative analysis on z‐stacks revealed that TF colocalizes for 7.9% ± 2.9% with G2‐DSA and for 7.6% ± 2.12% with G3‐DSA (*n* = 20 regions of interest from three cultures, Mann‐Whitney U test, *P* = 0.8881) (Figure 2E). While, CTX showed a colocalization of 32.7% ± 4.9% with G2‐DSA and 42.3% ± 4.36% with G3‐DSA (*n* = 20 regions of interest from three cultures, Mann‐Whitney U test, *P* = 0.2615) (Figure 2F). These data revealed uptake of DSA by both investigated mechanisms with no significant difference between G2‐DSA and G3‐DSA, but with a pronounced preference for the caveolae‐mediated endocytosis (for G2‐DSA = TF: 7.9% ± 2.9% vs CTX: 32.7% ± 4.9% *n* = 20 regions of interest from three cultures, Mann‐Whitney U test, *P* < 0.0007; for G3‐DSA = TF: 7.6 ± 2.12 % vs CTX: 42.3% ± 4.36% colocalization with G3‐DSA: *n* = 20 regions of interest from three cultures, Mann‐Whitney U test, *P* < 0.0001). In contrast to PAMAM dendrimers, the DSA do not follow only the clathrin‐mediated pathway, indicating that the dendrimer hybrids acquire different uptake properties compared to dendrimers alone.

### 2.2 DSA Are Efficiently Transported across the BBB In Vitro

**Figure 3:**
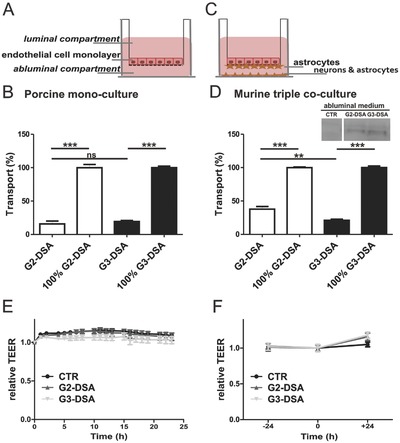
Transport of DSA in vitro. A,C) Schematic illustration of transwell assay with endothelial cells seeded in monoculture (A) or in triple coculture with astrocytes and neurons (C). B,D) Mean relative transport for DSA in porcine monoculture (B) or triple coculture (D). DSA (45 µg mL^−1^) were applied in the luminal compartment and transport was quantified relative to transport in insert without cells (100%). one‐way ANOVA, ***p < 0.001; **p < 0.01, ns = not significant. Porcine monoculture: G2‐DSA, 100% G2‐DSA *n* = 6 wells from three cultures in duplicate; G3‐DSA, 100% G3‐DSA *n* = 8 wells from four cultures in duplicate; triple coculture: *n* = 8 wells from four cultures in duplicate; one‐way ANOVA, ***p < 0.001; **p < 0.01, ns = not significant. (D inset) Streptavidin immunoblotting of abluminal medium. E,F) TEER monitoring of NP transport assay. TEER values were determined automatically during the whole experiment by CellZscope device (E) or at −24 h, 0 h, and +24 h from NP application by EVOM system (F). TEER values at *t* = 0 (DSA treatment) were set to 1 and each measurement expressed as relative value. Porcine monoculture: CTR *n* = 6 wells from three cultures in duplicate, G2‐DSA *n* = 6 wells from three cultures in duplicate, G3‐DSA *n* = 8 from four cultures in duplicate; TEER absolute values in a range between 50 and 300 Ω cm^2^. Triple coculture: *n* = 8 from four cultures in duplicate; TEER absolute values with a mean of 45 ± 13 Ω cm^2^.

Next, the transport of G2‐DSA and G3‐DSA from the luminal to the abluminal side of porcine and murine BBB transwell in vitro models was investigated. In one model porcine brain endothelial cells (PBECs) were seeded in the luminal side of transwell inserts in a monolayer system (**Figure** **3**A). The advantage of primary cells is that they keep features cell lines might have lost, like expression of efflux transporters and brain capillary specific enzymes important for the physiological function of the BBB. The porcine model was selected to obtain high transendothelial electrical resistance (TEER) values without the need of coculturing with astrocytes. Additionally, porcine genome, physiology, and anatomy reflect best the human biology compared to other laboratory animals.^34^, ^35^ To investigate the ability of DSA to cross the BBB, the percentage of transport was defined measuring the rhodamine fluorescence in the abluminal compartment after 24 h treatment. In monoculture, 15.7% ± 4.2% of G2‐DSA (*n* = 6 wells from three cultures in duplicate) compared to 100% G2‐DSA (100% ± 4.79, *n* = 6 wells from three cultures in duplicate, one‐way ANOVA, *P* < 0.0001) and 19.2% ± 1.56% of G3‐DSA (*n* = 8 wells from four cultures in duplicate) compared to 100% G3‐DSA (100% ± 2.29, *n* = 8 wells from four cultures in duplicate, one‐way ANOVA, *P* < 0.0001) were transported to the abluminal compartment (Figure 3B). A value of 100% represents the diffusion of DSA from luminal to abluminal compartment in inserts without cells. Comparing the two generations of dendrimers, no significant difference in transport rates could be observed (G2‐DSA: 15.7% ± 4.2% vs G3‐DSA: 19.2% ± 1.56%, *n* = 6–8 wells from three to four cultures in duplicate, one‐way ANOVA, *P* > 0.05).

Moreover, transport of DSA was validated in a murine triple coculture system using bEnd.3 as endothelial cell lines seeded on the luminal side of transwell inserts additionally to primary murine astrocytes in the abluminal side and primary murine neurons on the bottom of the abluminal compartment (Figure 3C). Since astrocytes and neurons are mainly responsible for the specialization of brain endothelial cells, the triple‐coculture system is the best in vitro approach to model the BBB.^36^ Using triple‐coculture, 37.8% ± 4.01% of G2‐DSA (*n* = 8 wells from four cultures in duplicate) compared to 100% G2‐DSA (100% ± 0.93, *n* = 8 wells from four cultures in duplicate, one‐way ANOVA, *P* < 0.0001) and 21% ± 1.69% of G3‐DSA (*n* = 8 wells from four cultures in duplicate) compared to 100% G3‐DSA (100% ± 2.3, *n* = 8 wells from four cultures in duplicate, one‐way ANOVA, *P* < 0.0001) were transported to the abluminal compartment (Figure 3D). Transport was also confirmed by detection of DSA‐streptavidin core in WB carried out on culture medium collected from the abluminal compartment after 24 h of DSA treatment (Figure 3D, inset). The triple coculture model showed a significantly higher transport for G2‐DSA compared to G3‐DSA (G2‐DSA: 37.8% ± 4.01% vs G3‐DSA: 21% ± 1.69%, *n* = 8 wells from four cultures in duplicate, one‐way ANOVA, *P* < 0.05), but it has to be considered that quantification of NP in the media did not take into account NP taken up by NVU cells in the abluminal compartment also involved in the uptake of DSA. Therefore, an underestimation of the real transport rate is expected since the total transport of DSA is constituted by DSA in abluminal medium + DSA taken up from cells seeded at the bottom of the abluminal compartment. In order to uncover whether this difference in the transport rate between the two discussed models is caused by the preferential cellular uptake of G3‐DSA, we next determined the estimated DSA fraction incorporated in cells (estimated DSA cellular fraction = DSA initial fluorescence – DSA abluminal fluorescence – DSA luminal fluorescence) (gray bar; Figure S1, Supporting Information). Intriguingly, we observed that for G3‐DSA in the triple coculture model a considerably higher fraction of fluorescence than monoculture vanished from the luminal and abluminal compartments (Figure S1A, Supporting Information), while for G2‐DSA the loss of fluorescence was constant and comparable between the two investigated BBB models (Figure S1B, Supporting Information). Indirectly, these results strongly suggest that a higher fraction of G3‐DSA than G2‐DSA is taken up by cells in triple coculture compared to monoculture. In summary, the data presented in this paragraph show that in both in vitro BBB systems, high transport efficiency for G2‐DSA and G3‐DSA from the luminal to the abluminal compartment could be observed. The comparison of DSA transport rate (in the range from 16% to 38%) and published in vitro transport rates highlights strongly the great potential of DSA to efficiently target the brain. Performing a comparison between different in vitro studies has to be carefully judged, because different experimental settings (e.g., the species of cells used, mono‐ and cocultures models) will be compared. In general, high transport rates have been demonstrated to be expected mainly in BBB models with lower permeability property^37^ as well as for nanosystems employing specific brain‐targeting ligands.^38^ However, for DSA the percentage of transport is higher compared to published rates of gold‐NP (conjugated to therapeutic agent and brain‐targeted peptide), transferrin receptor‐targeted immunoliposomes and neutral, anionic, and cationic malto‐dextrin 60 nm nanoparticles showing a percentage of transport which varies from 0.4% to 14%.^39^ Thus, the high in vitro transport efficiency for DSA shows very promising brain‐targeting properties of the investigated PAMAM dendrimer bioconjugates.

Using CellZscope for the porcine monolayer model and EVOM/Endhom chamber for the triple coculture, we measured the TEER before and after the transport to demonstrate that the used BBB models showed a tightness of the layer of brain endothelial cells high enough to consider transport data as reliable.^40^ DSA were applied when the absolute TEER values fell in a range between 50 and 300 Ω cm^2^ for porcine model or reached a mean value of 45 ± 13 Ω cm^2^ for triple coculture. The TEER was not influenced by treatment with DSA compared with *t* = 0 (time point of DSA application) (Figure 3E,F). In the triple coculture model the TEER even increased slightly during NP treatment for 24 h (Figure 3F). Hence, no paracellular leakage due to disruption of the BBB is to be expected. Tight junctions restrict paracellular diffusion of hydrophilic tracers. Compounds such as FITC‐dextran 4 KDa (FD4) can be used in order to monitor paracellular diffusion. The apparent permeability coefficient (*Papp*) for FD4 was calculated after measurement of FITC‐fluorescence in collected abluminal medium. The average basal *Papp* of FD4 was (9.24 ± 3.59) × 10^−7^ cm s^−1^ in our model employing PBECs and (1.28 ± 2.21) × 10^−6^ in triple coculture model. This falls within the range of the basal *Papp* values reported for FD4 in previously published in vitro BBB models.^41^ These validation experiments confirm the reliability of transport data, assuring that the shown efficiency of DSA to cross the BBB in vitro is not related to relevant paracellular transport.

### 2.3 DSA Follow a Transcytotic Pathway without Short‐Term Intracellular Degradation or Involvement of Autophagic Mechanisms

The transport of DSA from the luminal to the abluminal compartment of the transwell models implicates intracellular migration and trafficking which starts with the already discussed uptake. Multiple intracellular structures and organelles with their own identity and functions can be involved in trafficking and transcytocis. To clarify the molecular mechanisms of how DSA cross the BBB requires a deep investigation of the many interconnected intracellular pathways. The localization of DSA in cellular compartments was studied by using specific intracellular markers.

**Figure 4:**
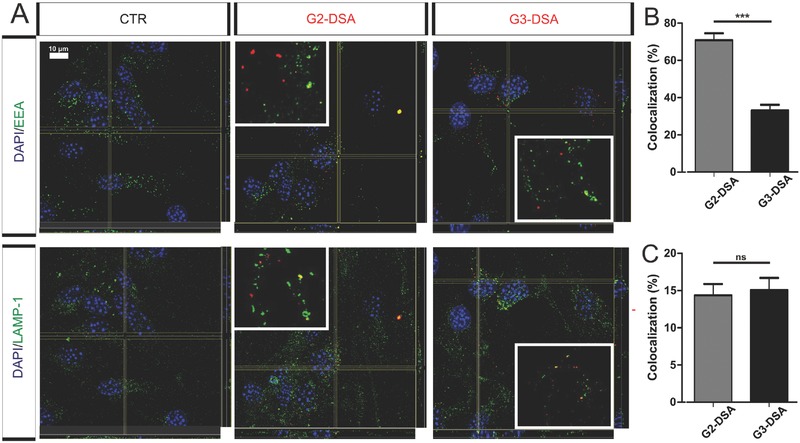
Endosomal trafficking of DSA in bEnd.3 cells. A) Confocal orthogonal view for colocalization of DSA‐positive vesicles (red) and early endosome antigen (EEA; green) for early endosomes, lysosomal‐associated membrane protein 1 (LAMP‐1; green) for late endosomes in bEnd.3 cells. Animated 3D reconstructions can be found in Videos 1–6. Insets: zoom of ROIs with colocalization (yellow). B,C) Quantification of colocalization for DSA with early endosomes (B) or late endosomes (C). Data are expressed in percentage of DSA‐positive‐vesicles partially colocalizing for at least 40 % with endosomal vesicles. *n* = 24 ROIs from three cultures; Mann‐Whitney U test, ***p < 0.001; ns = not significant.

Costaining with early endosome antigen (EEA) and lysosomal‐associated membrane protein 1 (LAMP‐1) were analyzed in order to investigate the possible localization of DSA respectively in early endosomes (EE) and late endosomes (LE). Representative orthogonal view and animated 3D reconstruction from z‐stacks revealed colocalization with both intracellular compartments in bEnd.3 cells after 24 h of DSA treatment (**Figure** **4**A and Videos 1–6). Quantification of colocalization of DSA with EE revealed significant generation‐dependent differences (G2‐DSA: 70.7% ± 3.73% vs G3‐DSA 33% ± 3.12%, *n* = 24 regions of interest from three cultures, Mann‐Whitney U test, *P* < 0.0001) (Figure 4B). By contrast, colocalization of DSA with LE showed a lower percentage than for EE and no difference between G2‐DSA and G3‐DSA (G2‐DSA: 14.3 ± 1.51 % vs G3‐DSA: 15% ± 1.62%, *n* = 24 regions of interest from three cultures, Mann‐Whitney U test, *P* = 0.7947) (Figure 4C). Short trafficking time in EE for G3‐DSA might be responsible for high uptake and transport rates.

Nonetheless, there were still NP containing vesicles that did not colocalize with EE or LE. In order to study in which other intracellular compartments DSA localized during trafficking, several other markers of vesicular transport were tested. First, using immunocytochemistry, we studied the possible colocalization of DSA with vesicle‐associated membrane protein 3 (VAMP3) that has been postulated to be a vesicle soluble N‐ethylmaleimide‐sensitive‐factor attachment receptor for early and mostly recycling endosomes, although mice with a null mutation in the encoding gene preserved intact endocytotic pathways.^42^ Tridimensional views obtained by confocal microscopy did not reveal a colocalization of DSA with VAMP3‐positive intracellular structures (Figure S2A, Supporting Information). Furthermore, DSA also did not colocalize with transferrin‐receptor (Figure S2B, Supporting Information). These data confirm the hypothesis that the PAMAM‐shell acquires new features in combination with a streptavidin core and that DSA do not preferentially follow a clathrin‐mediated pathway, in contrast to PAMAM dendrimers as discussed for couptake experiments.^33^

Previous findings demonstrated that some biodegradable NP can induce autophagy and be sequestered by autophagosomes.^43^ Thus, we further investigated the possible induction of autophagy and whether DSA localized in autophagosomes. Cultures of bEnd.3 cells were stained with anti‐microtubule‐associated protein 1A/1B‐light chain 3 (LC3), which is a marker for autophagosomes since, during autophagy, a cytosolic form of LC3 (LC3‐I) is conjugated to phosphatidylethanolamine to form an LC3–phosphatidylethanolamine conjugate (LC3‐II), which is recruited to autophagosomal membranes. Immunocytochemistry revealed that only very few NP‐containing vesicles might colocalize with autophagosomes (G2‐DSA: 0.8 ± 0.46 versus G3‐DSA 1.8% ± 0.86%, *n* = 17–20 regions of interest from three cultures; Mann‐Withney U test, *P* = 0.0244) (Figure S3A,B, Supporting Information).

Autophagy can lead to contrary cellular fates because it is involved in both beneficial and harmful cellular effects.^44^ While autophagy is activated at a basal level in most of the cells in the body with a role in regulating the turnover of long‐lived proteins and eliminating damaged structures, high levels of autophagy are often an indication of cellular stress. In order to address the question of whether DSA treatment might affect cellular homeostasis, induction of autophagy in bEnd.3 cells was monitored using immunocytochemical and biochemical approaches. First, comparing the ratio of LC3‐positive‐cells/total number of cells in control culture and NP‐treated samples, we demonstrated that DSA did not induce authophagy (CTR: 3.6% ± 0.44%; G2‐DSA: 2.8% ± 0.45%; G3‐DSA: 2.5% ± 0.35%, *n* = 30 regions of interest from three cultures, one‐way ANOVA, *P* = 0.1657) (Figure S3C, Supporting Information). Additionally, LC3‐II levels can be analyzed to define activation of authopagic processes, due to the fact that LC3‐II correlates with autophagosome numbers.^45^ To analyze the autophagic flux, we compared the measurement of the LC3‐II levels as a function of GAPDH (loading control) on WB. Bafilomycin A1 (BAF) was used to inhibit autophagosome–lysosome fusion to determine the activity of autophagic flux.^46^ No increase in LC3‐II levels was observed in bEnd.3 cells treated with DSA for 24 h (CTR + BAF: 1; G2‐DSA + BAF: 0.95 ± 0.14; G3‐DSA + BAF: 0.85 ± 0.03, *n* = 3 from three cultures, one‐way ANOVA, *P* = 0.4630) (Figure S3D,E, Supporting Information). Taken together, these data indicate no increase of autophagy in DSA‐treated cells with no incidence on the ratio of prosurvival/prodeath inputs.

Additionally, since endothelial cells show a relatively high basal autophagy,^47^ the detection of few NP‐containing‐autophagosomes suggests that DSA, in particular G3‐DSA given the high rate of uptake, might also be incorporated in autophagosomes during basal autophagy.

The data presented so far demonstrated the transport of DSA by transcytosis. It has to be considered that material taken up from outside the cell by endocytosis often is delivered to lysosomes and there degraded by acid hydrolases. In order to study if DSA might be temporarily delivered to lysosomes and partly degraded, we detected DSA by WB analysis in cell lysates after blocking the process of endosomes–lysosomes fusion with BAF (4 and 24 h treatment). We did not observe any change in levels of DSA uptake comparing BAF‐treated samples with untreated ones (G2‐DSA: 1, G2‐DSA + BAF 4 h: 1.04 ± 0.18, G3‐DSA: 1, G3‐DSA + BAF 4 h: 0.97 ± 0.06, G3‐DSA + BAF 24 h: 1.02 ± 0.01, *n* = 3 from three cultures, one‐way ANOVA, *P* = 0.9826) (Figure S4A,B, Supporting Information). These data suggest the involvement of a “proton‐sponge” mechanism as already proposed in previous studies for PAMAM dendrimers,^48^ which describes that cationic polymers have pH‐buffering properties inducing endosomal disruption. For this reason, PAMAM dendrimers exhibit high transfection efficiency compared to compounds without buffering ability.^49^ This fact offers the additional possibility of using DSA as a promising nanocarrier for nucleic acid and siRNA delivery preventing lysosomal degradation, one of the limiting steps for successful nucleic acid efficiency. In conclusion, the intracellular colocalization data presented herein confirm a transcytosis transport, which preserves DSA integrity and might involve lysosomal escape.

### 2.4 DSA Do Not Affect bEnd.3 Cells Viability and BBB Integrity

**Figure 5:**
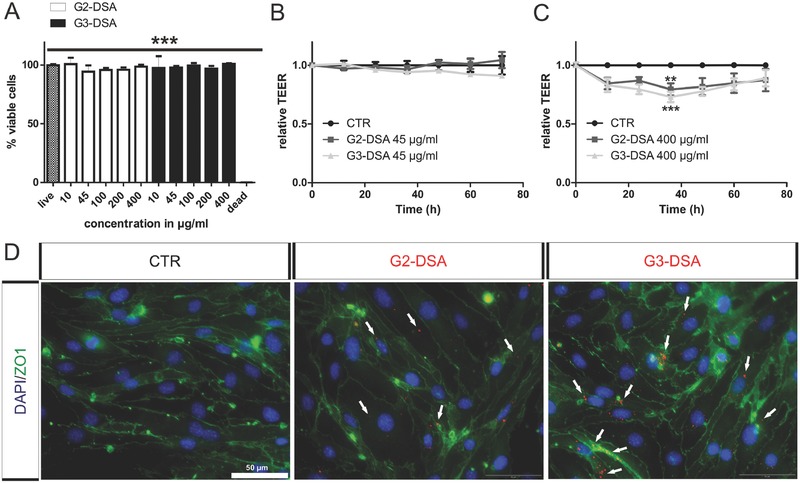
DSA effect on cell vitality and BBB integrity in vitro. A) bEnd.3 cell vitality by Alamar Blue assay after DSA treatment (24 h). Positive control: cell toxin staurosporine. *n* > 9 wells from four cultures; one‐way ANOVA; ***p < 0.001. B,C) Long term impact of DSA on cell integrity investigated for 72 h by ECIS. TEER values at *t* = 0 (DSA treatment) and TEER from CTR measurement were set to 1 and each measurement is expressed as a relative value. *n* (45 µg mL^−1^) = 5 wells from two cultures; *n* (400 µg mL^−1^) = 7 wells from three cultures, two‐way ANOVA, ***p < 0.001, **p < 0.01. D) Representative epifluorescence microscopy images from bEnd.3 cells culture treated with DSA (red) showing maintenance of BBB integrity by tight junctions staining with zona occludens 1 marker (ZO1; green). White arrows indicate representative areas of DSA localization.

Our nanosystem aims to target the brain without disruption or opening of the BBB and without affecting cell viability. Cell viability of bEnd.3 cells was challenged for 24 h with DSA (400 µg mL^−1^); applied concentration for uptake and transport studies was 45 µg mL^−1^. Cell vitality assay carried out by alamar blue showed no toxicity of the DSA for all concentrations (*n* > 9 wells from four cultures; one‐way ANOVA; *P* > 0.05). On the contrary, CTR and serial dilutions of DSA showed high significant differences with the staurosporine probe used as toxic control (live: 99.8% ± 0.75% vs dead: 1.81% ± 1.37%, *n* > 9 wells from four cultures, one‐way ANOVA, *P* < 0.05) (**Figure** **5**A).

Next, a concentration‐dependent impact of DSA on BBB integrity was probed. For this purpose, bEnd.3 cells were seeded in an ECIS 8‐Well chamber and TEER was measured continuously for up to 72 h after DSA application. The DSA concentration used in the previously discussed experiments (45 µg mL^−1^) showed no significant impact on the barrier integrity (*n* = 5 wells from two cultures, two‐way ANOVA, *P* > 0.05) (Figure 5B), while high concentrations of DSA (400 µg mL^−1^) have a low reversible impact on barrier integrity (Figure 5C). TEER values dropped to 79% for G2‐DSA (*n* = 7 wells from three cultures, two‐way ANOVA, *P* < 0.01) and 73% for G3‐DSA (*n* = 7 wells from three cultures, two‐way ANOVA, *P* < 0.001) within the first 40 h. However, barrier integrity recovered to 87% for G2‐DSA (*n* = 7 wells from three cultures, two‐way ANOVA, *P* > 0.05) and to 89% for G3‐DSA (*n* = 7 wells from three cultures, two‐way ANOVA, *P* > 0.05) at 72 h during DSA application. In agreement with previous studies and established therapies, a transient opening of the BBB does not have to be considered strictly as a deleterious event but could be considered as a system to better deliver molecules to the CNS.^50^ Additionally, zona occludens 1 (ZO1) staining as marker of tight junctions also show maintenance of intact tight monolayer in bEnd.3 cells treated for 24 h with DSA (45 µg mL^−1^) (Figure 5D). Thus, DSA represent a good biocompatible system which do not dramatically affect cell viability or BBB integrity.

### 2.5 DSA Uptake in Astrocytes and Neurons Occur without Cytotoxic Effects

**Figure 6:**
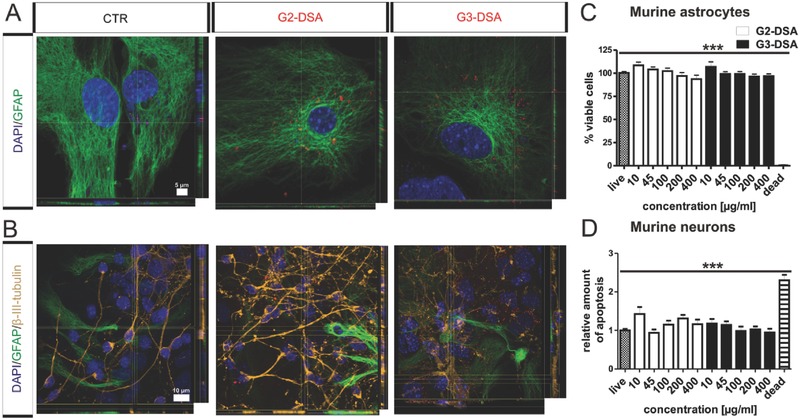
DSA uptake and cytotoxicity in target cells. A,B) Representative confocal 3D orthogonal views of DSA (red) uptake in GFAP‐positive astrocytes (green) A) and β‐tubulin‐positive neurons (orange) B). C,D) Cell vitality after DSA treatment (24 h) in astrocytes quantified by Alamar Blue assay (C) or in neurons by Fluorometric TUNEL assay (D). Positive control (dead): cell toxin staurosporine. Astrocytes: *n* > 9 from three cultures; neurons: *n* > 9 wells from three cultures; one‐way ANOVA; ***p < 0.001.

The aim of a successful brain treatment is not only to overcome the BBB, but also to reach target cells. These target cells in CNS diseases might be neurons or astrocytes. To investigate the potential of DSA to target NVU elements, primary cells were treated for 24 h, fixed and costained with markers for astrocytes (glial fibrillary acidic protein, GFAP) and neurons (ß‐III‐tubulin). High rate of uptake could be observed for both generations of DSA in glial cells (**Figure** **6**A), which represent the cell type coming in close contact with the tight layer of endothelial cells in BBB. In coculture of neurons and astrocytes, ß‐III‐tubulin stainings revealed uptake also in neuronal cells both in cell body and in dendrites as shown by colocalization of NP‐containing vesicles (Figure 6B).

As already observed for bEnd.3 cells, G3‐DSA showed higher uptake properties than G2‐DSA in all NVU cells. In addition, these data confirmed the previously mentioned hypothesis that lower G3‐DSA transport values in triple coculture system may result from a high uptake in target cells located in the abluminal compartment.

It has to be taken in account that delivery of exogenous molecules might cause side effects. Hence, in order to evaluate whether DSA affect cell viability of target cells, alamar blue assay for primary murine astrocytes and TUNEL assay for neurons were carried out, applying increasing concentrations of DSA from 10 up to 400 µg mL^−1^. After 24 h treatment, alamar blue assay showed no changes in astrocyte cell vitality for all the analyzed concentrations, even for DSA concentration up to 400 µg mL^−1^ (*n* > 9 from three cultures, one‐way ANOVA, *P* > 0.05) and significant differences were observed in comparison with a cytotoxic staurosporine treatment (live: 100.34% ± 1.4% vs dead: 0.5% ± 0.49%, *n* > 9 from three cultures, one‐way ANOVA, *P* < 0.05) (Figure 6C). Comparable results were obtained for primary neuronal cultures by TUNEL assay. There was no significant increase in apoptotic rate for DSA treated neurons (*n* > 9 from three cultures, one‐way ANOVA, *P* > 0.05) (Figure 6D), but a significant increase could be observed for the control treated with staurosporine (live: 1 ± 0.03 vs dead: 2.3 ± 0.14, *n* > 9 from three cultures, one‐way ANOVA, *P* < 0.05). These data revealed the ability of DSA to penetrate the NVU cells without induced cytotoxic effects.

### 2.6 DSA Are Transported to the Brain and Taken Up by NVU Cells In Vivo

Our in vitro studies showed the potential of DSA to cross the BBB, target neuronal cells and therefore potentially act as delivery system in clinical applications. The in vitro data presented so far demonstrated the importance of the dendrimer shell in promoting uptake and transport, which is dependent on the dendrimer‐generation most likely due to the amount of positive charges. Uptake and transport assays proved that G3‐DSA are more promising for further applications since the higher uptake in cells suggests higher efficiency in penetrating endothelial cells, crossing the BBB and more easily reaching target cells. For this reason, we focused the study on G3‐DSA to reach CNS after tail intravenous injection in mice. Coronal slices of 30 µm from brains isolated by CTR or Cy5‐labeled‐G3‐DSA treated mice were carefully analyzed and screened for DSA specific signal.

**Figure 7:**
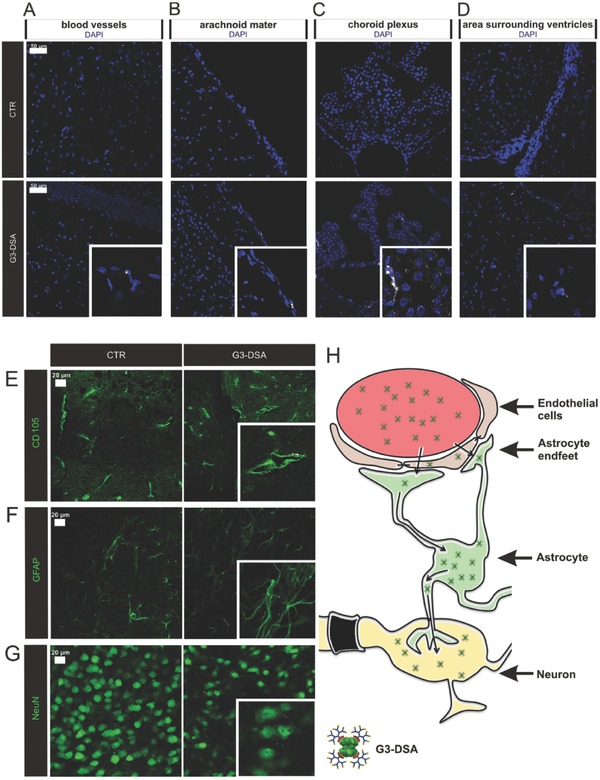
G3‐DSA transport in vivo. A–D) Representative confocal images of G3‐DSA (white) uptake at blood‐brain barriers and periventricular area 24 h after intravenous injection (450 µg mL^−1^ blood). E–G) Representative confocal images of G3‐DSA (white) uptake in brain endothelial cells (CD105; green) (E), astrocytes (GFAP; green) (F), and neurons (NeuN; green) (G) 24 h after intravenous injection (900 µg mL^−1^). H) Schematic representation of possible G3‐DSA in vivo trafficking.

Three main barrier layers are considered to separate the blood and the CNS: the endothelium of the brain microvessels, the epithelium of the choroid plexus, and the epithelium of the arachnoid mater.^51^ Uptake of G3‐DSA was easily detectable in cells surrounding blood vessels (**Figure** **7**A). G3‐DSA were also clearly taken up in the meninges in correspondence of the second barrier layer represented by the epithelium of the arachnoid mater (Figure 7B). However, highest uptake was observed in the choroid plexus and ventricles, suggesting that G3‐DSA were released into the cerebrospinal fluid as well (Figure 7C,D). These three barriers represent the way through which our DSA can access the brain. Therefore, it is not surprising that G3‐DSA were more easily detectable in areas surrounding the ventricles as consequence of the high uptake in that specific brain area (Figure 7D). Uptake of G3‐DSA into the brain was also confirmed by WB analysis on brain lysate. On a PVDF membrane, clear bands for DSA were visible after detection of streptavidin core (Figure S5, Supporting Information). To more specifically identify the localization of DSA within the brain tissue, G3‐DSA signal was detected in combination with staining for endothelial cells using the marker endoglin (CD105), glia using the marker GFAP, or neurons using the marker NeuN. CD105 staining clearly showed G3‐DSA uptake on the lumen side of the vessels as well as in the abluminal brain parenchyma (Figure 7E). Lower intensity DSA signals were also detected in target cells: astrocytes (Figure 7F) and neurons (Figure 7G). These results indicate an effective migration of bioconjugates from blood to brain which might follow the hypothesis of permeation from endothelial cells to neurons mediated by astrocytes whose endfeet take up DSA by surrounding the endothelium of blood vessels (Figure 7H).

A comparable mechanism might be responsible for the migration of pure PAMAM dendrimers through the corpus callosum one‐week post intracranial injection described by Srinageshwar et al.^52^ In order to quantify the amount of G3‐DSA reaching the brain after intravenous injection, the percentage of injected dose (% ID) as well as the concentration of G3‐DSA expressed in µg/g of brain tissue was calculated. The results show that 0.03% ± 0.0% ID (*n* = 3) and 0.44% ± 0.03 µg g^−1^ of brain tissue (*n* = 3) of G3‐DSA efficiently overcome the BBB and reach the brain in vivo. The obtained transport values are in the range of known dendrimer systems^24^, ^53^ and show for example a tenfold higher efficiency of transport to the brain in healthy animals compared to a published PAMAM G4‐ hydroxyl‐terminated dendrimer.^24^ One of the crucial points in the study presented here is the BBB penetration in healthy animals across an intact barrier. Indeed, most of already published studies tend to evaluate transport efficiency for in vivo disease models in neurological disorders, which are often accompanied by BBB impairment. This leads to an overestimation of the actual crossing ability of the used nanosystem due to increased BBB permeability in pathological conditions, if transport is not additionally determined in healthy animals. In our study, demonstrating high transport efficiency across BBB in healthy animals, we can envision even a higher transport rate if the BBB is affected by the neurological disorders or in brain cancer, which treatment is addressed to. Additionally, the observed G3‐DSA brain concentration is in a range, which would reach the needed concentration for therapeutic molecules like nucleic acids. For example, Tyler et al. demonstrated a decrease in gene expression of neurotensin receptor using peptide nucleic acids at a brain molecular concentration almost fivefold lower than G3‐DSA.^54^ Nance et al. found a % ID of 0.003% (healthy animal) for G4‐OH PAMAM dendrimer loaded with N‐acetylcysteine to efficiently deliver the drug load in the diseased animal with 100‐fold greater brain accumulation compared to free drug.^26^ Additionally, from observed data it could be supposed that DSA might protect drug load from lysosomal entrapment holding the possibility of even higher efficiency.

### 2.7 DSA Are Biodistributed in Many Organs In Vivo, Mostly in Kidney and Liver

Although this study aims to investigate DSA transport to the brain, additionally the uptake of G3‐DSA in other organs was investigated. Therefore, after each NP application, kidney, liver, spleen, lung, and heart were also collected and screened for NP uptake. G3‐DSA showed high uptake rates in kidney and liver (Figure S6A,B, Supporting Information). NP signal was also detected in the spleen, lung, and heart with lower uptake rates (Figure S6C–E, Supporting Information).

Considering the physicochemical properties of DSA, small size in the range of 5 nm and positively charged, a high uptake in kidney was predictable.^55^

The present data demonstrate high kidney uptake of DSA, which could indicate a clearance or renal reabsorption. To further investigate the case for DSA, WB analysis on blood samples collected 24 h after G3‐DSA treatment revealed a circulation of the nanocompound indicating that NP were still available for uptake (Figure S7, Supporting Information).

### 2.8 DSA Do Not Affect BBB Integrity In Vivo

**Figure 8:**
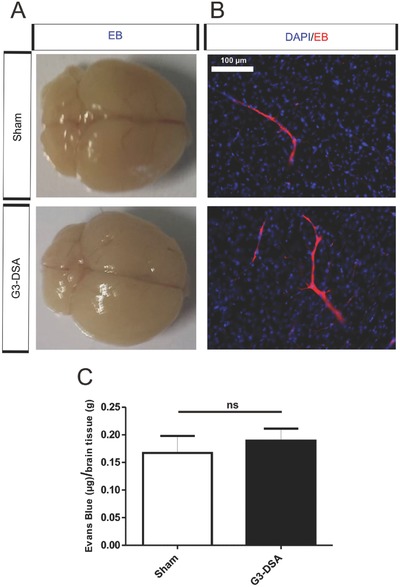
G3‐DSA effect on BBB integrity in vivo. A) Representative pictures of intact brains showing absence of Evans blue (EB) extravasation in sham as well as G3‐DSA‐treated mice after 24 h from the injection. B) Representative epifluorescence microscopy images from brain slices showing presence of EB (red) in blood vessels without permeation in brain parenchyma in sham and G3‐DSA‐treated mice. C) EB quantification in brain lysate from sham and G3‐DSA‐treated mice. Data are expressed as µg of EB per g of brain tissue. *n* = 3 brain lysates from three mice; Mann‐Whitney U test; ns = not significant.

To assess the use of DSA for medical applications, the BBB integrity was studied by the use of intravital dyes (tracers, markers) of molecular weight greater than 180 Da, which preclude passage across an intact barrier. EB (MW 961 Da) is one of the largest dyes, which in blood binds to the albumin fraction (EBA) to give rise to a high‐molecular complex (EBA = 68 500 Da).^56^ EB was intravenously injected 3 h before the end of DSA treatment to assess BBB integrity. Analysis of the brains did not show macroscopic EB infiltration in brain tissue related to DSA treatment (**Figure** **8**A). Epifluorescence microscopy revealed a localized EB fluorescence in blood vessels without extravasation of the dye (Figure 8B). In addition, analysis of brain lysate (measuring the µg of EB g^−1^ of brain tissue) did not show significant change of EB fluorescence compared to the sham animal (Sham: 0.17 ± 0.03 µg g^−1^ of brain tissue versus G3‐DSA: 0.19 ± 0.02 µg g^−1^ of brain tissue, *n* = 3 brain lysates from three mice, Mann‐Whitney U test, *P* = 0.6905) (Figure 8C). This data exclude a correlation between in vivo G3‐DSA uptake and BBB disruption and proves the biocompatibility of our demdrimer protein bioconjugate.

## 3 Conclusion

Combining in vitro and in vivo approaches by using BBB models, NVU cell cultures as well as intravenous injection and analysis on tissue of mice, we demonstrated that DSA (I) are taken up by neurovascular unit cells, (II) are transported from the bloodstream to the brain via transcytosis, (III) are biocompatible for NVU cells, and (IV) do not impair BBB integrity.

Showing for the first time a biocompatible and multifunctional PAMAM dendrimer bioconjugate which can efficiently target the brain via crossing the intact BBB, significantly expands the existing potential of dendrimer‐based drug delivery systems to may be translated into future trials involving CNS pharmacotherapies. Additionally, the DSA itself might be applicable as antiviral, antitoxin, and antiprion therapeutic agent suitable for therapy of CNS infections or it could be tuned for the delivery of various therapeutics or diagnostic tracers into the brain. In a previous study, Ng et al.^57^ successfully demonstrated that the tetrameric streptavidin adapter represents a robust scaffold for the attachment of biotinylated biological active therapeutic molecules.

We envision transporting endogenous molecules or growth factors, to complement endogenous mechanism for neuronal protection and repair. The observed high uptake into astrocytes could have potential for treating reactive gliosis occurring in brain traumata, multiple sclerosis and Alzheimer's disease, by loading DSA with nerve growth factor (NGF) as NGF has been already shown to be antigliosis and neuroprotective.^58^ Nonetheless, brain targeted chemotherapeutics may also represent a possible suitable drug cargo for glioma or brain tumor in general. Additionally, DSA could serve as a platform for gene therapy approaches. The lysosomal escape mechanism related to the cationic nature of DSA points to the possible delivery of nucleic acids, which could compensate gene mutations often related to neurological disorders. In case of prion diseases, a group of fatal neurodegenerative disorders in human and animals,^59^ PAMAM dendrimers with high surface density of amino groups have been shown to be effective in inhibiting prion proteins, whose accumulation in CNS is associated to neurological dysfunction.^16^, ^30^ Cationic PAMAM dendrimers are also described to inhibit pore formation by binary anthrax toxin (PA63) and Clostridium botulinum C2 toxin (C2IIa), thereby protecting HeLa and Vero cells from intoxication.^17^ Thus, the possibility of implementing DSA as therapeutics for treatment of prion diseases or bacterial intoxication could be envisioned. Furthermore, DSA provide the potential to combine intrinsic therapeutic properties of PAMAM dendrimers with therapeutic molecules in one system.

## 4 Experimental Section

*Ethical Approval*: All experimental procedures were approved by the ethical committee of the “Landesuntersuchungsamt Rheinland‐Pfalz” and the authority “Landesuntersuchungsamt Rheinland‐Pfalz” protocol number: “Aktenzeichen “23 177‐07/G 16‐1‐024.” Principles of laboratory animal care (European, national and international laws) were followed.

*Materials*: All reagents were obtained from Sigma‐Aldrich (Seelze or Hamburg, Germany), unless otherwise specified, of analytical grade and used as received. Streptavidin from Streptomyces avidinii was bought from HiSS Dianostics GmbH and was used as is. Vivaspin 6 ultrafiltration tubes (10 kD MWCO) were purchased from Sartorius. Water was purified by a Milli‐Q filter system.

The chemical synthesis of generation 2 (G2) and generation 3 (G3) PAMAM dendron and subsequent biotinylation was reproduced exactly according to the literature.^57^, ^60^

*Rhodamine Labeled Streptavidin*: Streptavidin (2 mg, 0.038 µmol) was dissolved in PBS buffer (2 mL, pH 7.4) and lissamine sulfonyl chloride (22 µL, 1 mg mL^−1^ stock, 0.038 µmol) predissolved in DMSO was added and stirred overnight. The reaction solution was purified using Vivaspin 6 ultrafiltration tube (10 kD MWCO) with ×3 H_2_O washes and the concentrated salt‐free solution was lyophilized to afford rhodamine labeled streptavidin in 90% yield.

*Cyanine 5 Labeled Streptavidin*: Streptavidin (2 mg, 0.038 µmol) was dissolved in PBS buffer (2 mL, pH 7.4) and added cyanine‐5 NHS ester (6.5 µg, 0.0076 µmol) (Biomol GmbH, Hamburg, Germany). The reaction solution was shaken overnight on an orbital shaker purified using Sephadex G25M (GE Healthcare) size exclusion chromatography. The purified fraction was collected and lyophilized to afford the product in 92% yield.

*Dendronization of Rhodamine/Cy5 Labeled Streptavidin*: Rhodamine/Cy5 labeled streptavidin (1 mg, 0.019 µmol) was dissolved in PBS buffer (1 mL, pH 7.4) and added to the respective biotinylated dendron (0.094 µmol). The mixture was shaken for 3 h on an orbital shaker and purified using Sephadex G25M (GE Healthcare) size exclusion chromatography. The fluorescent fractions were collected and analyzed. Analysis of the complex was performed on an FPLC (ÄKTA purifier, GE Healthcare), 200 mm Superose 6 size exclusion column with tris buffer (50 × 10^−3^ m, pH 9.0) as the eluent (*R*_v_ = 14 mL). The corresponding bands containing the dendronized protein was identified and lyophilized.

*DSA Uptake*: DSA were added to cell culture medium (final concentration of 45 µg mL^−1^) for 24 h. For couptake studies DSA were applied in combination to transferrin‐Alexa Fluor 488 (T13342, Invitrogen, 120 µg mL^−1^ ) or cholera toxin B subunit‐FITC (C1655, 15 µg mL^−1^).

*Transport Assay*: Transport of DSA was investigated in an in vitro transwell assay. Cells were seeded in monoculture or triple coculture system on BD Fluoroblok TM Inserts (0.3 cm^2^, pore size 3 µm, Corning Incorporated, Corning, USA). DSA (45 µg mL^−1^) were luminal applied. Crossing rate was quantified due to rhodamine labeling of NP. Fluorescence was measured with an Infinite F1000 TECAN plate reader and the percentage of crossing was calculated. Fluorescence intensity in abluminal compartment of transwell system without cells was set to 100%. The estimated DSA cellular fraction was obtained as follows: estimated cellular fraction = DSA initial fluorescence – DSA abluminal fluorescence – DSA luminal fluorescence.

*Measurement of Transendothelial Electrical Resistance*: TEER in the in vitro triple coculture system was determined using an EVOM voltohmmeter with an Endohm chamber for 6 mm culture cups (World Precision Instruments, Berlin). The final TEER value (Ω × cm^2^) was calculated from measured ohm (*Ω*) subtracted by the *Ω* values of insert without cells, multiplied by insert surface area of 0.3 cm^2^. TEER and capacitance of the cell layer (Ccl) of a PBECs monolayer on the transwell insert was automatically determined by CellZscope system (NanoAnalytics, Münster, Germany). When TEER started to increase, culture medium was replaced by EBM‐2 containing supplements (rhFGF‐B, rhEGF, GA‐100, Ascorbic Acid, R^3^‐ICF‐1, Heparin), hydrocortisone (550 × 10^−9^ m), pCPT‐cAMP (C3912, 250 × 10^−6^ m), and RO 20–1724 (Calbiochem; 557502, 17.5 × 10^−6^ m) to induce TEER values.^34^, ^61^ This BBB model was used for transport and permeability experiments.

TEER of a surface attached bEnd.3 monolayer was determined using an Electrical Cell Impedance Sensing array (ibidi in cooperation with Applied BioPhysics, Martinsried, Germany). Cells were seeded in an 8‐well chamber (ECIS culture ware 8W10E, ibidi in cooperation with Applied BioPhysics, Martinsried, Germany) and were challenged with DSA, when cells reached maximal plateau impedance values. For all the just described methods, TEER values at time point 0 h were set to 1 and data expressed as relative values.

*Viability Assay*: Cytotoxity on bEnd.3 cells and astrocytes was investigated using the cell viability Alamar Blue assay. Cells were DSA‐treated for 24 h after reaching confluence. For viability quantification cells were washed with HBSS−/−. Resazurin (R7017, 16.66 µg mL^−1^) in HBSS+++ (100 µL) was added to each well. After 1 h incubation, fluorescence was measured spectrophotometrically using a Tecan Infinite F1000 plate reader (Tecan, Salzburg, Austria). Fluorescence values were corrected to the absorbance of resazurin solution without cells (blank). Cell viability (%) was related to untreated control cells (100%).

Cytotoxicity for primary neurons was quantified using a fluorimetric TUNEL assay (ROCHE, DNA Fragmentation Imaging Kit, 06432344001, Mannheim, Germany), which allows simultaneous quantification of cell numbers and apoptotic cells. On DIV 9 cells were challenged with DSA for 24 h. To quantify the relative amount of total and apoptotic cells the DNA Fragmentation Imaging Kit was used. The relative fluorometric TUNEL values were measured with an Infinite F1000 TECAN plate reader and relative values for cell numbers and ratio of all groups were normalized to untreated control cells. As positive control cells were treated with staurosporine (1 × 10^−3^ m). All experiments were run at least three times in triplicates.

*NP In Vivo Studies*: Cy5‐labeled‐DSA (450 µg mL^−1^ blood; or 900 µg mL^−1^ blood for NP‐positive cells determination and in vivo quantification) were administered via the tail vein in P21 mice. CTR mice were PBS injected. After 24 h, mice were perfused with PBS supplemented with heparin‐natrium (Ratiopharm, 5000 E.I.) and subsequently with PFA 4%. Brain was collected as well as liver, kidney, spleen, lung, and heart. To study in vivo BBB integrity a solution of 2% Evans blue (E2129) was administered i.v. for 3 h. To quantify in vivo brain uptake, mice were injected with G3‐DSA. After 24 h treatment, perfusion with PBS/heparin was carried out and brains were weighted, homogenized by TissueRuptor (Qiagen) in cell lysate, and incubated for 3 h. Subsequently, brain lysates were sonicated (5 cycles of 30 s) and centrifuged (4000 × g, 10 min). Cy5‐fluorescence for G3‐DSA was measured by Tecan Infinite F1000 plate reader. % ID and µg/g of brain tissue of transported G3‐DSA were calculated referring the fluorescence values to a calibration curve obtained via serial dilutions of Cy5‐DSA in brain lysates (modified protocol from Nance et al.).^24^

*In Vivo Organ Preparation*: Organs were equilibrated in 30% sucrose and 30 µm slices were obtained using a freezing microtome (Leica CM 1325). Mice to study the BBB integrity after Evans blue administration were perfused with PBS/heparin and one hemisphere was incubated in TCA (8789, Roth) 50%. Brains in TCA were then smashed by TissueRuptor (Qiagen), centrifuged for 25 min at 12000 × g. Supernatant was collected and loaded in a 96‐well‐plate for EB fluorescence measurement by Infinite F1000 TECAN plate reader. EB (µg)/brain tissue (g) was calculated referring to a calibration curve in a range from 0 to 1 µg mL^−1^ of EB in TCA.

To generate brain lysate, after treatment and perfusion with PBS/heparin (Ratiopharm), the collected brain was smashed in lysis buffer by TissueRuptor (Qiagen). The homogenate was incubated on a rotation wheel 3 h at 4 °C and then centrifuged for 5 min at 2000 × g. Supernatant was collected and used for WB analysis. For EB histochemistry detection brains were collected fresh without perfusion.

*Image Analysis*: Images were taken by an IX81 microscope and a monochrome fluorescence CCD camera XM10 using the cell^F Software (Olympus) or by a TCS SP5 confocal (Leica). For quantification intracellular colocalization, z‐stacks were deconvolved by Huygens Essential software. For uptake quantification in ImageJ fixed thresholds were applied for DAPI and rhodamin signal and fluorescence positive particles were counted for each image. DSA positive vesicles per cell are blotted. For colocalization studies the percentage of vesicles containing DSA colocalizing with the specific intracellular compartment was calculated. A threshold of 40% of colocalization for each vesicle was set to avoid false positive values. In addition, deconvolved z‐stacks were also analyzed by JacoP plugin in ImageJ to calculate Van Steensel's crosscorrelation functions as proof of colocalization data.^62^

*Data Analysis*: All data were analyzed with GraphPad Prism 5 software (Graph Pad, La Jolla, CA, USA) and were presented as mean ± SEM. Data were analyzed with the Mann‐Whitney U test, ANOVA one‐way and ANOVA two‐way with post hoc test. *P* < 0.05 was considered as statistically significant *, *P* < 0.01 **, *P* < 0.001 ***.

## Conflict of Interest

The authors declare no conflict of interest.

## Supporting information

Supplementary

Supplementary

Supplementary

Supplementary

Supplementary

Supplementary

Supplementary

## References

[1] K. T. Thakur , E. Albanese , P. Giannakopoulos , N. Jette , M. Linde , M. J. Prince , T. J. Steiner , in Disease Control Priorities: Mental, Neurological, and Substance Use Disorders, 3rd ed., Vol. 4, (Eds: Patel V., Chisholm D., Dua T., Laxminarayanm R., Medina‐Mora M. E.) World Bank, Washington, DC 2016, p. 87.

[2] M. S. Kinch , Drug Discovery Today 2015, 20, 1040. 25681791 10.1016/j.drudis.2015.02.003

[3] B. T. Hawkins , T. P. Davis , Pharmacol. Rev. 2005, 57, 173. 15914466 10.1124/pr.57.2.4

[4] W. M. Pardridge , Drug Discovery Today 2007, 12, 54. 17198973 10.1016/j.drudis.2006.10.013

[5] W. M. Pardridge , NeuroRx 2005, 2, 3.15717053

[6] M. A. Bellavance , M. Blanchette , D. Fortin , AAPS J. 2008, 10, 166. 18446517 10.1208/s12248-008-9018-7 PMC2751463

[7] A. B. Etame , R. J. Diaz , C. A. Smith , T. G. Mainprize , K. Hynynen , J. T. Rutka , Neurosurg. Focus 2012, 32, E3. 10.3171/2011.10.FOCUS11252 PMC4106119 22208896

[8] A. M. Cook , K. D. Mieure , R. D. Owen , A. B. Pesaturo , J. Hatton , Pharmacotherapy 2009, 29, 832. 19558257 10.1592/phco.29.7.832

[9] a) E. J. Kwon , M. Skalak , R. Lo Bu , S. N. Bhatia , ACS Nano 2016, 10, 7926; 27429164 10.1021/acsnano.6b03858 PMC5896006

[10] D. J. Begley , M. W. Brightman , Prog. Drug Res. 2003, 61, 39. 14674608 10.1007/978-3-0348-8049-7_2

[11] W. A. Banks , Nat. Rev. Drug Discovery 2016, 15, 275. 26794270 10.1038/nrd.2015.21

[12] J. Kreuter , Adv. Drug Delivery Rev. 2014, 71, 2. 10.1016/j.addr.2013.08.008 23981489

[13] J. Barar , M. A. Rafi , M. M. Pourseif , Y. Omidi , Bioimpacts 2016, 6, 225. 28265539 10.15171/bi.2016.30 PMC5326671

[14] V. Leiro , S. Duque Santos , C. D. F. Lopes , A. Paula Pêgo .

[15] J. M. McCarthy , B. Rasines Moreno , D. Filippini , H. Komber , M. Maly , M. Cernescu , B. Brutschy , D. Appelhans , M. S. Rogers , Biomacromolecules 2013, 14, 27. 23234313 10.1021/bm301165u

[16] Y. B. Lim , C. E. Mays , Y. Kim , W. B. Titlow , C. Ryou , Biomaterials 2010, 31, 2025. 20022103 10.1016/j.biomaterials.2009.11.085

[17] P. Förstner , F. Bayer , N. Kalu , S. Felsen , C. Förtsch , A. Aloufi , D. Y. Ng , T. Weil , E. M. Nestorovich , H. Barth , Biomacromolecules 2014, 15, 2461. 24954629 10.1021/bm500328v PMC4215879

[18] O. Klementieva , E. Aso , D. Filippini , N. Benseny‐Cases , M. Carmona , S. Juvés , D. Appelhans , J. Cladera , I. Ferrer , Biomacromolecules 2013, 14, 3570. 24004423 10.1021/bm400948z

[19] R. Huang , H. Ma , Y. Guo , S. Liu , Y. Kuang , K. Shao , J. Li , Y. Liu , L. Han , S. Huang , S. An , L. Ye , J. Lou , C. Jiang , Pharm. Res. 2013, 30, 2549. 23703371 10.1007/s11095-013-1005-8

[20] I. Posadas , B. López‐Hernández , M. I. Clemente , J. L. Jiménez , P. Ortega , J. de la Mata , R. Gómez , M. A. Muñoz‐Fernández , V. Ceña , Pharm. Res. 2009, 26, 1181. 19191011 10.1007/s11095-009-9839-9

[21] N. H. Gamage , L. Jing , M. J. Worsham , M. M. Ali , J. Nanomed. Nanotechnol. 2016, 7, 393. 27699139 10.4172/2157-7439.1000393 PMC5040461

[22] M. J. Serramía , S. Álvarez , E. Fuentes‐Paniagua , M. I. Clemente , J. Sánchez‐Nieves , R. Gómez , J. de la Mata , M. Muñoz‐Fernández , J. Controlled Release 2015, 200, 60. 10.1016/j.jconrel.2014.12.042 25559178

[23] H. Dai , R. S. Navath , B. Balakrishnan , B. R. Guru , M. K. Mishra , R. Romero , R. M. Kannan , S. Kannan , Nanomedicine 2010, 5, 1317. 21128716 10.2217/nnm.10.89 PMC3095441

[24] E. Nance , F. Zhang , M. K. Mishra , Z. Zhang , S. P. Kambhampati , R. M. Kannan , S. Kannan , Biomaterials 2016, 101, 96. 27267631 10.1016/j.biomaterials.2016.05.044 PMC5379995

[25] F. Zhang , J. Trent Magruder , Y. A. Lin , T. C. Crawford , J. C. Grimm , C. M. Sciortino , M. A. Wilson , M. E. Blue , S. Kannan , M. V. Johnston , W. A. Baumgartner , R. M. Kannan , J. Controlled Release 2017, 249, 173. 10.1016/j.jconrel.2017.01.032 PMC5323327 28137632

[26] E. Nance , S. P. Kambhampati , E. S. Smith , Z. Zhang , F. Zhang , S. Singh , M. V. Johnston , K. Rangaramanujam , M. E. Blue , S. Kannan , J. Neuroinflammation 2017, 14, 252. 29258545 10.1186/s12974-017-1004-5 PMC5735803

[27] S. Mignani , M. Bryszewska , M. Zablocka , B. Klajnert‐Maculewicz , J. Cladera , D. Shcharbin , J.‐P. Majoral , Prog. Polym. Sci. 2017, 64, 23.

[28] A. Gothwal , P. Kesharwani , U. Gupta , I. Khan , M. C. Iqbal Mohd Amin , S. Banerjee , A. K. Iyer , Curr. Pharm. Des. 2015, 21, 4519. 26311317 10.2174/1381612820666150827094341

[29] V. Leiro , J. P. Garcia , H. Tomás , A. P. Pêgo , Bioconjugate Chem. 2015, 26, 1182. 10.1021/bc5006224 25826129

[30] S. Supattapone , H. O. Nguyen , F. E. Cohen , S. B. Prusiner , M. R. Scott , Proc. Natl. Acad. Sci. USA 1999, 96, 14529. 10588739 10.1073/pnas.96.25.14529 PMC24470

[31] F. Zhang , P. Mastorakos , M. K. Mishra , A. Mangraviti , L. Hwang , J. Zhou , J. Hanes , H. Brem , A. Olivi , B. Tyler , R. M. Kannan , Biomaterials 2015, 52, 507. 25818456 10.1016/j.biomaterials.2015.02.053 PMC4710089

[32] a) J. Voigt , J. Christensen , V. P. Shastri , Proc. Natl. Acad. Sci. USA 2014, 111, 2942; 24516167 10.1073/pnas.1322356111 PMC3939899

[33] L. Albertazzi , M. Serresi , A. Albanese , F. Beltram , Mol. Pharm. 2010, 7, 680. 20394437 10.1021/mp9002464

[34] A. Patabendige , R. A. Skinner , L. Morgan , N. J. Abbott , Brain Res. 2013, 1521, 16. 23603406 10.1016/j.brainres.2013.04.006 PMC3694295

[35] Y. Zhang , C. S. Li , Y. Ye , K. Johnson , J. Poe , S. Johnson , W. Bobrowski , R. Garrido , C. Madhu , Drug Metab. Dispos. 2006, 34, 1935. 16896068 10.1124/dmd.105.006437

[36] T. Ruck , S. Bittner , S. G. Meuth , Neural Regener. Res. 2015, 10, 889. 10.4103/1673-5374.158342 PMC4498345 26199600

[37] D. Liu , B. Lin , W. Shao , Z. Zhu , T. Ji , C. Yang , ACS Appl. Mater. Interfaces 2014, 6, 2131. 24417514 10.1021/am405219u

[38] A. J. Clark , M. E. Davis , Proc. Natl. Acad. Sci. USA 2015, 112, 12486. 26392563 10.1073/pnas.1517048112 PMC4603510

[39] a) L. Fenart , A. Casanova , B. Dehouck , C. Duhem , S. Slupek , R. Cecchelli , D. Betbeder , J. Pharmacol. Exp. Ther. 1999, 291, 1017; 10565819

[40] K. Benson , S. Cramer , H. J. Galla , Fluids Barriers CNS 2013, 10, 5. 23305242 10.1186/2045-8118-10-5 PMC3560213

[41] P. J. Gaillard , A. G. de Boer , Eur. J. Pharm. Sci. 2000, 12, 95. 11102736 10.1016/s0928-0987(00)00152-4

[42] a) C. Hu , D. Hardee , F. Minnear , Exp. Cell Res. 2007, 313, 3198; 17651732 10.1016/j.yexcr.2007.06.008 PMC2696983

[43] J. Zhang , X. Zhang , G. Liu , D. Chang , X. Liang , X. Zhu , W. Tao , L. Mei , Theranostics 2016, 6, 2099. 27698943 10.7150/thno.16587 PMC5039683

[44] a) S. J. Cherra , C. T. Chu , Future Neurol. 2008, 3, 309; 18806889 10.2217/14796708.3.3.309 PMC2544613

[45] a) B. Loos , A. du Toit , J. H. Hofmeyr , Autophagy 2014, 10, 2087; 25484088 10.4161/15548627.2014.973338 PMC4502790

[46] C. Mauvezin , T. P. Neufeld , Autophagy 2015, 11, 1437. 26156798 10.1080/15548627.2015.1066957 PMC4590655

[47] T. Urbanek , W. Kuczmik , A. Basta‐Kaim , B. Gabryel , Brain Res. 2014, 1553, 1. 24462935 10.1016/j.brainres.2014.01.017

[48] W. Liang , J. K. W. Lam in Molecular Regulation of Endocytosis (Eds: Ceresa B.), InTech, London, UK 2012, Ch. 17.

[49] a) L. Zhou , L. Gan , H. Li , X. Yang , J. Pharm. Biomed. Anal. 2007, 43, 330; 16872783 10.1016/j.jpba.2006.06.021

[50] J. J. Choi , S. Wang , T. R. Brown , S. A. Small , K. E. Duff , E. E. Konofagou , Ultrason. Imaging 2008, 30, 189. 19149463 10.1177/016173460803000304 PMC3919133

[51] N. J. Abbott , in Drug Delivery to the Brain: Physiological Concepts, Methodologies and Approaches (Eds: Hammarlund‐Udenaes M., de Lange E. C. M., Thorne R. G.), Springer, New York 2014, p. 3.

[52] B. Srinageshwar , S. Peruzzaro , M. Andrews , K. Johnson , A. Hietpas , B. Clark , C. McGuire , E. Petersen , J. Kippe , A. Stewart , O. Lossia , A. Al‐Gharaibeh , A. Antcliff , R. Culver , D. Swanson , G. Dunbar , A. Sharma , J. Rossignol , Int. J. Mol. Sci. 2017, 18, E628. 28335421 10.3390/ijms18030628 PMC5372641

[53] W. Ke , K. Shao , R. Huang , L. Han , Y. Liu , J. Li , Y. Kuang , L. Ye , J. Lou , C. Jiang , Biomaterials 2009, 30, 6976. 19765819 10.1016/j.biomaterials.2009.08.049

[54] B. M. Tyler , K. Jansen , D. J. McCormick , C. L. Douglas , M. Boules , J. A. Stewart , L. Zhao , B. Lacy , B. Cusack , A. Fauq , E. Richelson , Proc. Natl. Acad. Sci. USA 1999, 96, 7053. 10359837 10.1073/pnas.96.12.7053 PMC22053

[55] E. Blanco , H. Shen , M. Ferrari , Nat. Biotechnol. 2015, 33, 941. 26348965 10.1038/nbt.3330 PMC4978509

[56] P. Kozler , J. Pokorný , Physiol. Res. 2003, 52, 607. 14535837

[57] D. Y. Ng , J. Fahrer , Y. Wu , K. Eisele , S. L. Kuan , H. Barth , T. Weil , Adv. Healthcare Mater. 2013, 2, 1620. 10.1002/adhm.201200419 23657926

[58] A. M. Colangelo , L. Alberghina , M. Papa , Neurosci. Lett. 2014, 565, 59. 24457173 10.1016/j.neulet.2014.01.014

[59] S. B. Prusiner , Proc. Natl. Acad. Sci. USA 1998, 95, 13363. 9811807 10.1073/pnas.95.23.13363 PMC33918

[60] S. L. Kuan , B. Stöckle , J. Reichenwallner , D. Y. Ng , Y. Wu , M. Doroshenko , K. Koynov , D. Hinderberger , K. Müllen , T. Weil , Biomacromolecules 2013, 14, 367. 23210662 10.1021/bm301531c

[61] L. L. Rubin , D. E. Hall , S. Porter , K. Barbu , C. Cannon , H. C. Horner , M. Janatpour , C. W. Liaw , K. Manning , J. Morales , J. Cell Biol. 1991, 115, 1725. 1661734 10.1083/jcb.115.6.1725 PMC2289219

[62] S. Bolte , F. P. Cordelières , J. Microsc. 2006, 224, 213. 17210054 10.1111/j.1365-2818.2006.01706.x

