# Supplementary material for: Brain Delivery of Multifunctional Dendrimer Protein Bioconjugates
Source: Adv Sci (Weinh). 2018 Feb 23;5(5):1700897. doi: 10.1002/advs.201700897 (PMC5979778; doi:10.1002/advs.201700897)
Supplement: Supplementary file 1 — Supplementary [file ADVS-5-1700897-s002.pdf]

Copyright WILEY-VCH Verlag GmbH & Co. KGaA, 69469 Weinheim, Germany, 2018.

Supporting Information

for *Adv. Sci.,* DOI: 10.1002/advs.201700897

Brain Delivery of Multifunctional Dendrimer Protein Bioconjugates

*Pierpaolo Moscariello, David Y. W. Ng, Malin Jansen, Tanja Weil, Heiko J. Luhmann, and Jana Hedrich**

Supporting Information

Title: Brain Delivery of Multifunctional Dendrimer Protein Bioconjugates.

Pierpaolo Moscariello, David Y. W. Ng, Malin Jansen, Tanja Weil, Heiko J. Luhmann, Jana Hedrich[*]

Material and Methods

Culturing bEnd.3 Cells: The murine line, bEnd.3, from brain endothelioma (American Type Culture Collection, Manassas, VA, USA) was cultured as recommended by the manufacturer.(48) Cultures were maintained at a humidified atmosphere with 37°C and 5 % CO2. Media consisted of DMEM (Glutamax, gibco by life technology, Darmstadt, Germany) supplemented with 10 % fetal calf serum (Biochrom, S0115) and 2 % penicillin/streptomycin (Invitrogen GmbH, Karlsruhe, Germany). bEnd.3 passages 10–30 were used for experimental assays. Cells were seeded with 80.000 cells per insert (0.3 cm2) or per well in 96-well-plate, 100.000 cells per coverslip, 100.600 cells per ECIS-8-well-chamber.

Primary Murine Astrocytes: Primary astrocyte cultures were generated from six mice (P3). Forebrains were collected and meninges were removed, then the tissue was minced and incubated with 0.05 % trypsin/EDTA for 20 min. Digestion was stopped with 10 % horse serum (HS; S9135, Biochrom) in HBSS -/- (gibco by life technology, Darmstadt, Germany). Cells were dispersed using Pasteur pipettes and filtered by a 40 µm cell strainer. Cells were seeded on poly-ornithine (P4538) coated T75-flasks in culture medium (MEM supplemented with 10 % horse serum and 5 µg mL-1 gentamycin). Cells were subcultured by splitting the cells 1:3. Astrocytes were seeded with 100.000 cells/coverslip, 52.000 cells/well in IBIDI-8-Well-Chamber, 25.000 cell/insert (0.3 cm2).

Primary Murine Neurons: Primary neuronal dissociated cell culture from mice were performed as introduced by Kaech and Banker(49) and optimized by the protocol of Beaudoin and colleagues.(50) Forebrains were collected from mice pups (P0-1) and meninges were removed. Tissue was washed three times with ice cold HBSS -/- and incubated for 20 min in 0.05 % Trypsin/EDTA (gibco by life technology, Darmstadt, Germany). DNase I (Roche, 11284932001) (2000U in 5 mL) was added for 5 min. After 3 washing steps tissue was homogenized in Neuronal Plating Medium (MEM, 10 % horse serum, 0.6 % glucose) and stained with trypan blue (T8154) for cell counting. Primary neurons were seeded with cell numbers of 60.000 cells/well in 96-well-plates or 200.000 cells/coverslip (SDS treated and poly-ornithine coated) in plating medium. After 30 min medium was replaced with Neuronal Maintenance Medium [Neurobasal Medium (gibco by life technology, Darmstadt, Germany) with 2 x 10-3 M Glutamine (gibco by life technology, 25030024) and supplemented with B27 Supplement (gibco, 17504044)]. Cells were fed every 7 days by replacing one-third of the medium with fresh Neuronal Maintenance Medium.

Triple Co-Culture: For the in vitro triple co-culture model primary astrocytes were seeded on day 0 with a cell number of 25.000 cells/insert on the abluminal side of the permeable transwell membrane and 25.000 cells/coverslip. Neurons were grown in Neuronal Maintenance Media on coverslips in separate wells. On day 2-3 bEnd.3 cells (passage 10-30) were seeded with 80.000 cells/insert in the luminal compartment. Co-culture was grown in EBM-2-Basal-Medium (Lonza, Walkersville, USA) supplemented with + 15 % FCS, Glutamine (0.863 mg mL-1) and EBM-2 Single Quot Kit without VEGF growth factor. TEER of co-culture was monitored and the quality benchmark for usage in vitro transport assay was a TEER of at least 27.5 Ωcm2 on day 9-11. On Day 9-11 of co-culture and DIV 7-9 of neuronal culture inserts were transferred to wells with neurons on the bottom, TEER was measured before and 24 hours after DSA treatment.

Isolation of Porcine Brain Endothelial Cells (PBECs): Porcine brains were collected from butcher Färber in Alzey, Germany and transported on ice in transport buffer (10 % HEPES, 1 % Pen/strep, 1 % Ciprobay 200 Bayer, 1 % Fungizone). Primary brain microvascular endothelial cells were generated as described previously by Freese et al..[45] Two brain hemispheres were incubated 1 h on ice in incubation buffer (10 % HEPES, 1 % Pen/Strep, 1 % Ciprofloxacin Kabi 400 mg/200 mg, 1 % Fungizone). Meninges were peeled off and the gray matter was collected in PBS, minced and centrifuged at 1400 rpm for 10 min at 4°C. Each hemisphere was digested for 30 min at 37°C in DMEM containing 20 mg of Collagenase IV (Worthington; LS004210) and 20 µg of DNase I in a total volume of 3 mL. After washing the tissue was resuspended in a solution of 20 % Percoll (GE Healthcare; 17-0891-01) diluted in PBS and centrifuged at 2600 rpm for 1 h at 4°C. The capillary fragments from both hemispheres were digested for 10 min at 37°C in DMEM containing 2 mg of Collagenase/Dispase (Roche, 10269638061) and 20 µg of DNase I in a total volume of 2 mL and filtered by 100 µm cell strainer. The cell suspension was loaded on a Percoll gradient previously prepared (24 mL Percoll + 13 mL PBS + 2 mL 10x PBS centrifuged at 10000 x g for 2 h at room temperature). The brain endothelial cell fraction was resuspended in EBM-2 medium (Lonza; CC-3156) containing supplements (rhFGF-B, rhEGF, GA-100, Ascorbic Acid, R3-ICF-1, Heparin, Hydrocortisone; Lonza; CC-4114A), 15 % FCS and of Glutamine (0.863 mg mL-1). Cells were seeded in transwell inserts or coverslips precoated with fibronectin (F1141, 5 µg mL-1). Finally, PBECs selection was carried out by puromycin (Enzo; BML-GR312-0050) treatment. At DIV 0 cells were treated with of puromycin (4 µg mL-1, at DIV 1 with 3 µg mL-1 and 2 µg mL-1 at DIV 2).

Determination of Apparent permeability (Papp): was determined using FITC-Dextran 4 (FD4) (46944). FD4 (100 µg mL^{-1}) was applied to the luminal side of the transwell system. After 24 h

medium from the abluminal compartment was collected and FD4 fluorescence intensity was measured by Infinite F1000 TECAN plate reader. FD4 concentration was determined in respect to a calibration curve obtained by the measurement of serial dilutions values and the apparent BBB permeability coefficient was calculated according to Artursson et al. 1990,[46] by the following equation 1:

$Papp$ = dQ/dt * 1/A*C_0 [cm s^{-1}].

dQ/dt is the amount of transported FITC-dextran per minute (µg sec^{-1}.), A is the surface area of the filter (0.3 cm^2), C_0 is the initial FITC-dextran concentration (100 µg mL^{-1}).

Immunocytochemistry and Histochemistry: Cells or organs were fixed with PFA 4 %. Probes were blocked and permeabilized with 7 % normal donkey serum (Dianova, Hamburg, Germany)/ 0.3 % (0.8 % for organ slices) Triton (T-8787) in PBS for 2 h at RT. Organ slices were at first incubated with donkey anti-mouse FAB fragment (Dianova, 715-007-003, Hamburg, Germany) in PBS 10 x 10^{-3} M for 2 h at RT. Primary antibody was incubated in 2 % bovine serum albumin (Dianova, 001-000-161, Hamburg, Germany) with 0.05 % azide and 0.1 % (0.3 % for organ slices) Triton/PBS overnight at RT. Secondary antibody and DAPI (32670) were incubated in 2 % bovine serum albumin with 0.05 % azide for 2 h at RT, followed by PBS washing. Organ slices were additionally washed with Tris-HCl (50 x 10^{-3} M) at a pH of 6.5. Cells or slices were finally embedded in Fluoromount (Biozol, Eching, Germany) or CFM-3R (Citifluor Ltd., London, UK).

Table1. Antibodys for immunohistochemistry

**Primary antibody**	**Secondary antibody**
**ZO-1**<br>anti-zonula occludens 1 (rabbit, Zymed via invitrogen, 61-300, 1:100)	Cy2 goat anti-rabbit IgG (Dianova, 111-25-144, 1:10)
**EEA**<br>anti-early endosome antigen-1 (rabbit,	Alexa Fluor 647 donkey anti-rabbit IgG (Jackson, Dianova, 711-605-152, 1:200)

Abcam, ab50313, 1:50)	
**LAMP1** anti-cluster of differentiation 107a (rat, BD Biosciences, 553792, 1:50)	DyLight 488 anti-rat IgG (Bethyl Lab, Biomol, 1:200)
**VAMP3** anti-vesicle-associated membrane protein 3 (rabbit, Synaptic System, 1:250)	Cy2 donkey anti-rabbit IgG (Jackson, Dianova, 711-225-152, 1:200)
**TfR** anti-transferrin receptor (mouse, Zymed via Invitrogen, 1:500)	Dylight 488 donkey anti-mouse IgG (Bethyl Lab, Biomol, A90-337D2, 1:200)
**LC3** anti-microtubule-associated protein 1A/1B-light chain 3 (mouse, Nanotools, 0260S/LC3-2G6, 1:200)	Dylight 488 donkey anti-mouse IgG (Bethyl Lab, Biomol, A90-337D2, 1:200)
**GFAP** anti-glial fibrillary acidic protein (mouse, Synaptic Systems, 173011)	Dylight 488 donkey anti-mouse IgG (Bethyl Lab, Biomol, A90-337D2, 1:200)
**NeuN** anti-neuronal nuclear antigen (rabbit, Merck Millipore, ABN78)	Dylight 488 donkey anti-rabbit IgG (Jackson, Dianova, 711-485-152, 1:200)
**CD105** anti-endoglin (rat, Beckman Coulter, 732334)	DyLight 488 anti-rat IgG (Bethyl Lab, Biomol, 1:200)

Protein Concentration: Culture medium after transport assay from abluminal compartment was centrifuged at 14000 x g using Amicon Ultra 10K centrifugal filter devices (UFC501024; Millipore) to concentrate the protein in a volume of 20 µL according to the provided protocol.

C*ell Lysate:* bEnd.3 cells were scraped off in culture medium and the suspension was centrifuged for 5 min at 4°C at 200 x g. The pellet was washed with PBS and centrifuged for 5 min at 4°C at 400 x g, dissolved in 1 volume of lysis buffer (50 x 10^{-3} M Tris; 150 x 10^{-3} M NaCl; 1 x 10^{-3} M EDTA; 1 % Triton-X-100) supplemented with protease inhibitor (Roche) and incubated 45 min on rotation wheel at 4°C. The cell lysate was obtained collecting the supernatant after centrifugation for 5 min at 4°C at 13000 x g. The pellet was also dissolved in 1 volume of lysis buffer to obtain the cell pellet probe. Cell lysate and cell pellet probes were stored at -20°C.

Western Blot Analysis: Samples were separated by electrophoresis on a SDS-Polyacrylamide gel and transferred onto a polyvinylidene difluoride membrane. The membrane was blocked with non-fat 4 % milk in TBST (500 x 10^{-3} M Tris base; 1.5 M NaCl; pH 7.2; 0.1 % Tween 20). Antibodies against streptavidin (ab10020; 1:500; Abcam), LC3 (mab LC3-2G6; 1:200; Nanotools) or GAPDH (A300-641A, 1:5000, Bethyl Laboratories) diluted in non-fat 4 % milk in TBST were applied. Finally, the protein of interest was detected by HRP-conjugated anti-mouse antibody (115-035-003; 1:5000; Dianova) or HRP-conjugated anti-rabbit antibody (111-035-144; 1:10000; Dianova) and visualized by ECL Western Blotting substrate (Merck Millipore) according to the provided protocol.

Analysis of Autophagy Induction by LC3-II Immunoblotting: bEnd.3 cells seeded in 24 well/plate were treated for 24 h with DSA (45 µg mL^{-1}). In the last 4 h of the 24 h treatment period, cells were incubated with BAF (400 x 10^{-9} M) to inhibit autophagosome/lysosome fusion. Cell lysates were then used for WB detection of endogenous LC3-II.

Analysis of DSA Short-Term Lysosomal Degradation: bEnd.3 cells seeded in 24 well/plate were treated for 24 h with of DSA (45 µg mL^{-1}). A concentration of BAF (400 x 10^{-9} M) was applied for 24 h in co-treatment with DSA or in the last 4 h of the DSA treatment to inhibit lysosomal entrapment and degradation of DSA. Cell lysates were then used for WB detection of streptavidin core.

Supplementary Figures:

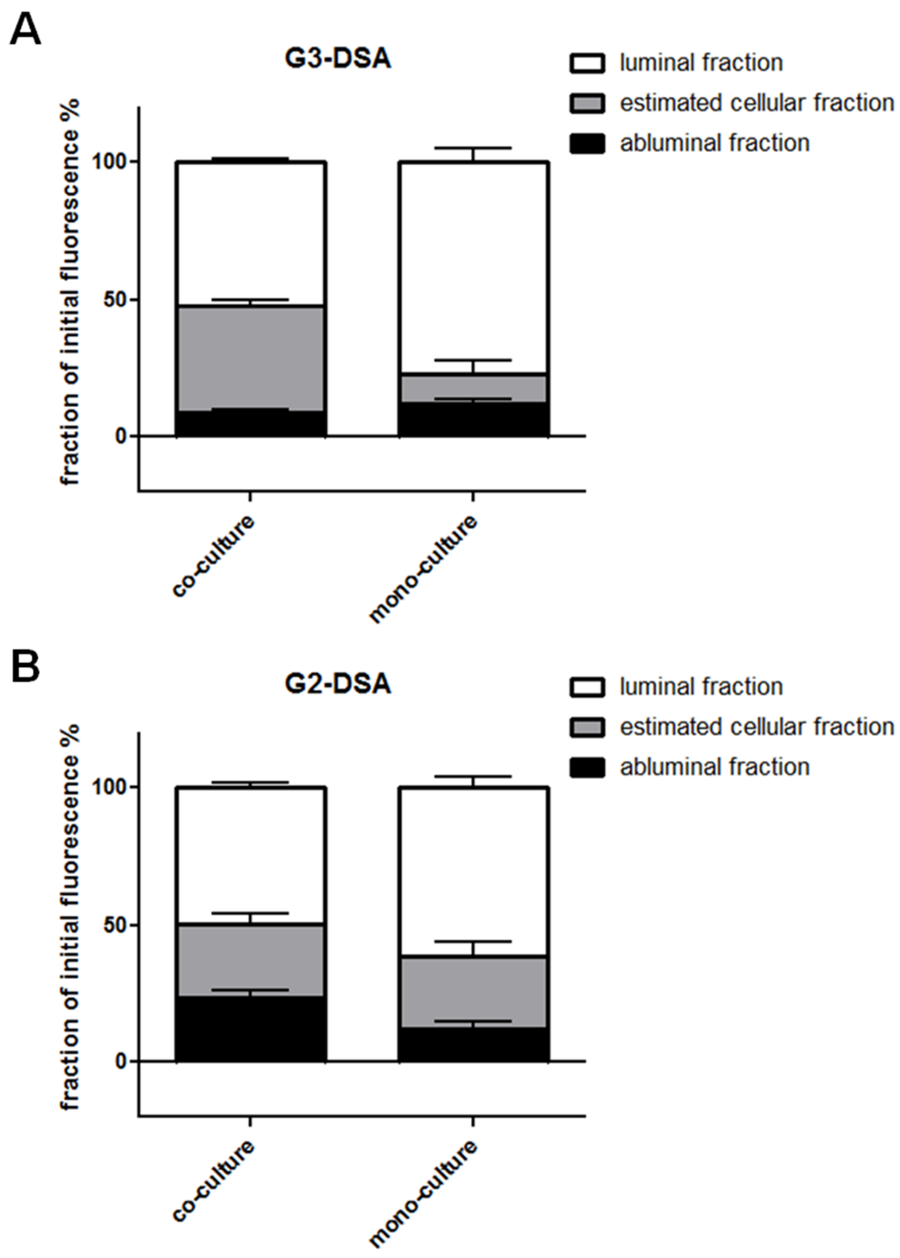

Figure S1. DSA fractions of BBB in vitro models. Three different fractions of fluorescence in the transwell model are blotted. After 24 h of G3-DSA **(A)** or G2-DSA **(B)** application in luminal compartment. Estimated cellular fraction = DSA initial fluorescence - DSA abluminal fluorescence - DSA luminal fluorescence. Co-culture: n = 8; mono-culture: n = 6.

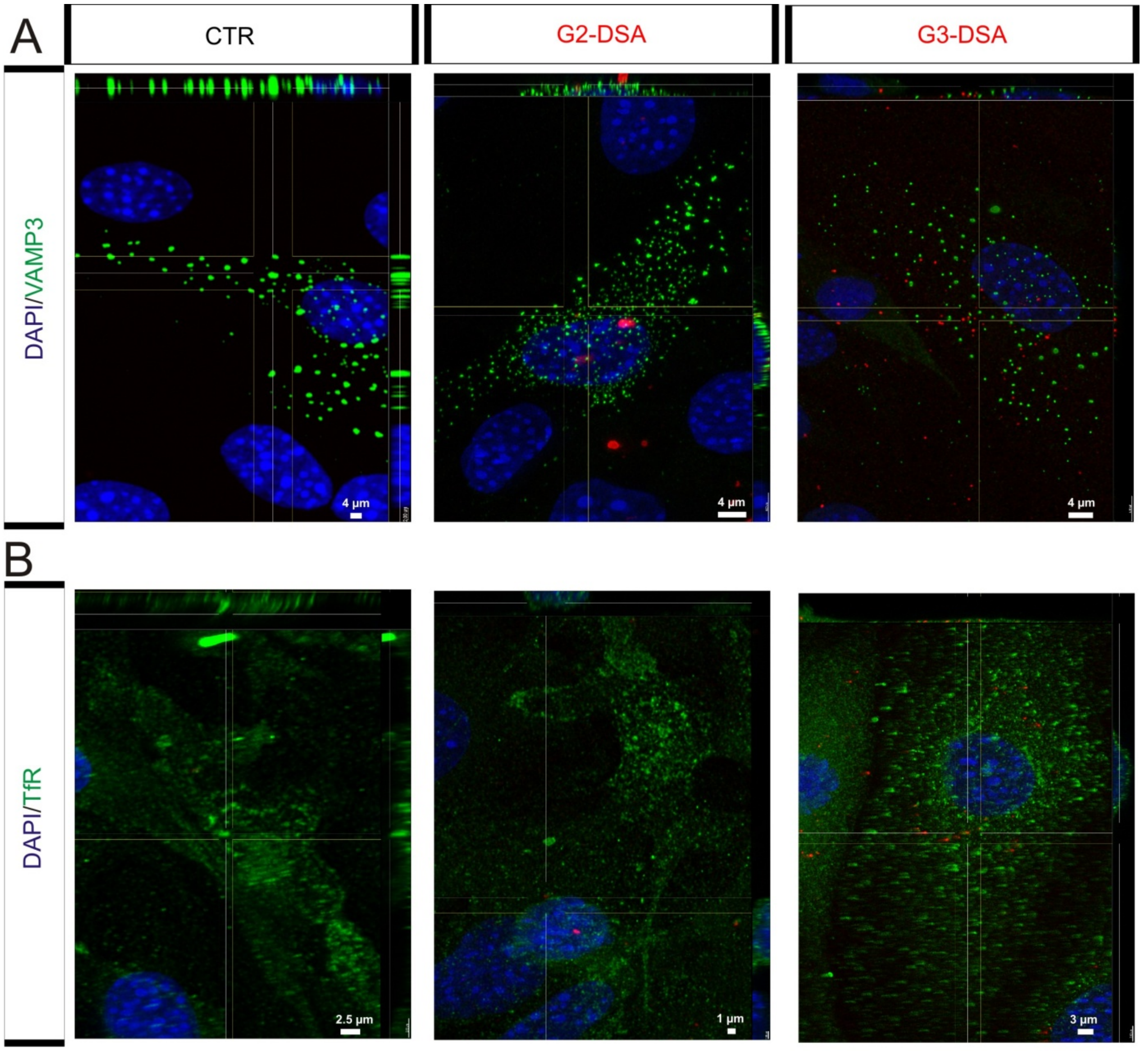

Figure S2. DSA intracellular trafficking. Representative confocal tridimensional orthogonal view for colocalization analysis of DSA-positive vesicles (red) and vesicle associated membrane protein 3 (VAMP3; green) **(A)** for recycling endosomes or transferrin receptor (TfR; green) **(B)**.

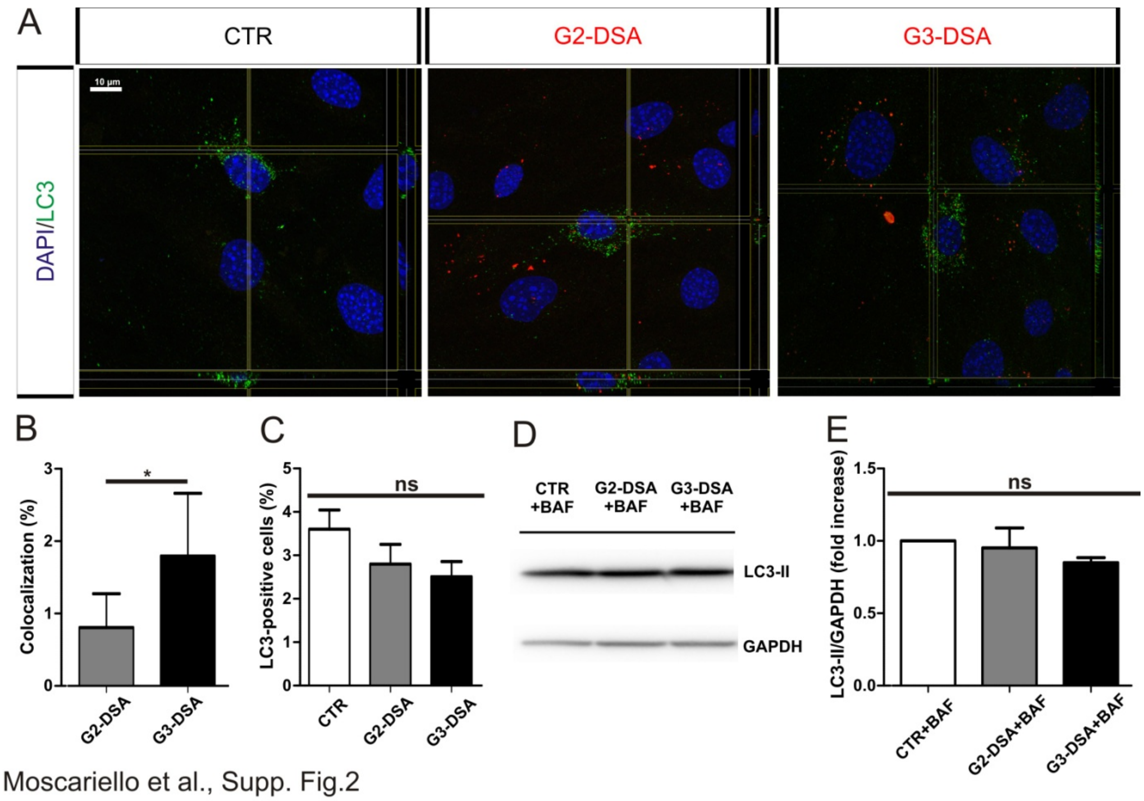

Figure S3. Effect of DSA on autophagy. (A) Representative confocal tridimensional orthogonal view for colocalization analysis of DSA-positive vesicles (red) and microtubule-associated protein 1A/1B-light chain 3 (LC3; green). **(B)** Quantification of colocalization for DSA with autophagosomes. Data are expressed in percentage of DSA-positive-vesicles partially colocalizing for at least 40 % with autophagosome-vesicles. G2-DSA n = 20 ROIs, G3-DSA n = 17 ROIs from 3 cultures; Mann-Whitney U test, *p < 0.05. **(C)** Percentage of LC3-positive cells in CTR or DSA treated bEnd.3 cells. Total number of cells in each analyzed ROI represents 100%. n = 30 ROIs from 3 cultures; one-way ANOVA; ns = not significant. **(D, E)** Western blot of lysates **(D)** and bands densitometric quantification **(E)** of LC3-II levels in CTR and DSA-treated cells. Bafilomycin (BAF) was used as inhibitor of autophagosome-lysosome fusion.n = 3 cell lysates from 3 cultures; one-way ANOVA; ns = not significant.

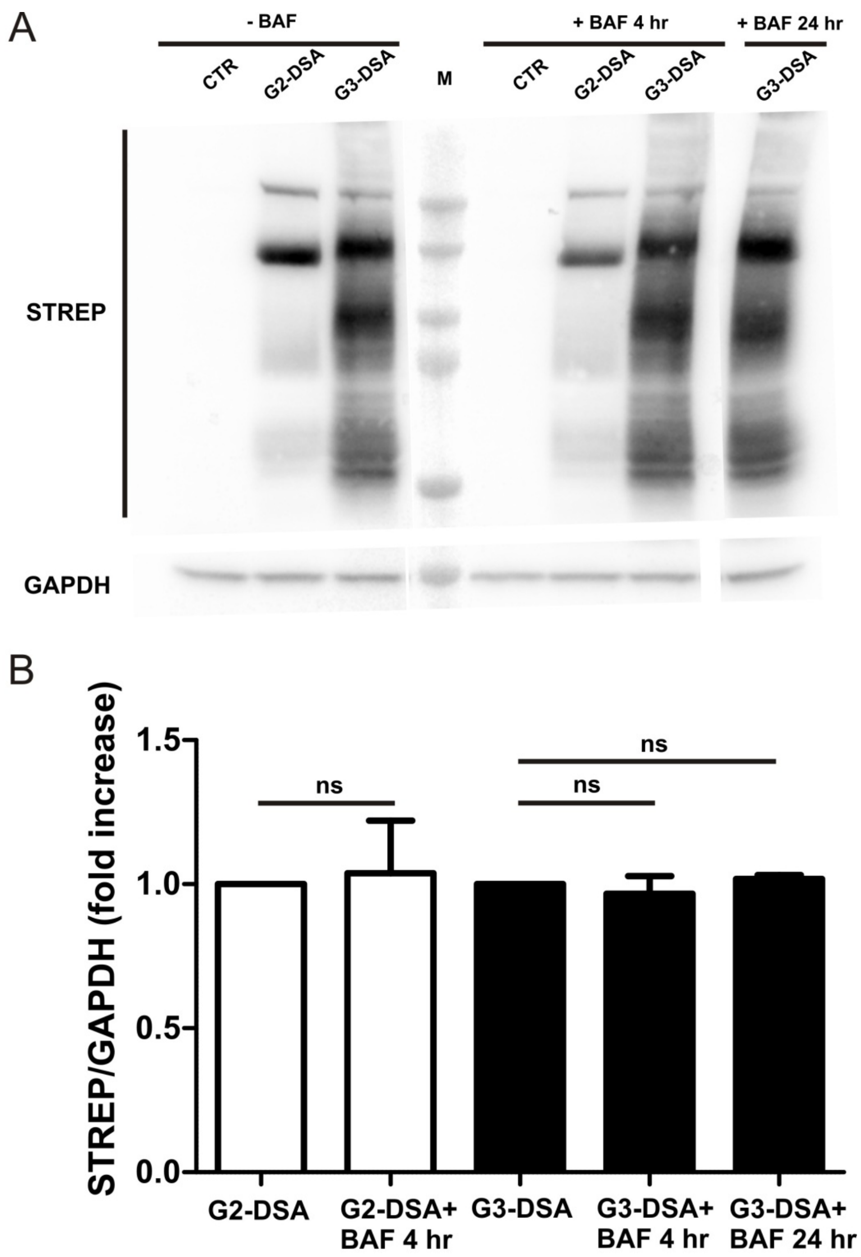

Figure S4. DSA lysosomal degradation. (A) Streptavidin (STREP) immunoblotting of lysate from CTR and DSA treated bEnd.3 cells. Bafilomycin (BAF) treatment for 4 h or 24 h was used to induce lysosomal escape of DSA. **(B)** Densitometric quantification of STREP levels for DSA-treated cells with or without BAF treatment. STREP levels of not BAF-treated samples were set to 1 and each correspondent BAF-treatment expressed as relative value; n = 3 cell lysates from 3 cultures; one-way ANOVA; ns = not significant.

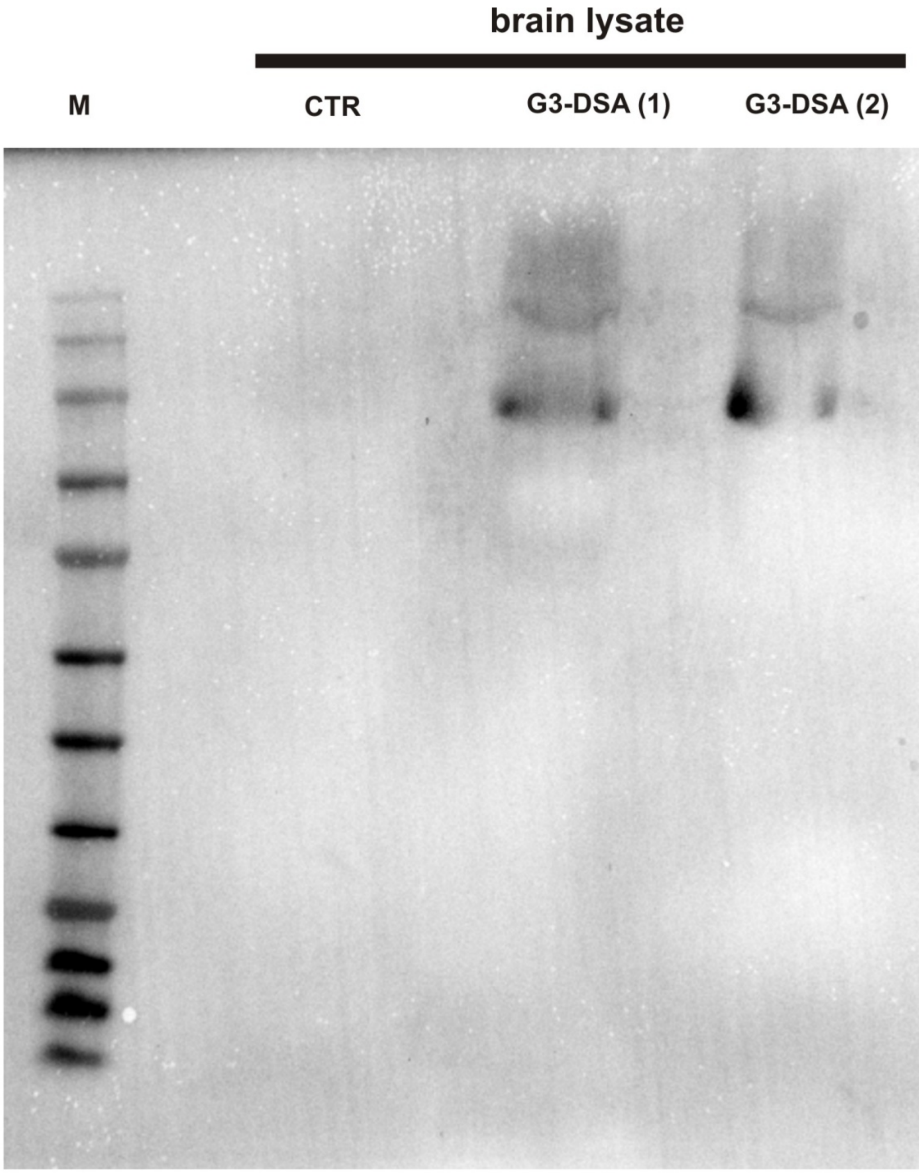

Figure S5. G3-DSA brain uptake in vivo. Streptavidin immunoblotting of brain lysate from CTR and 2 x G3-DSA treated mice (450 µg mL^{-1}) 24 h post intravenous injection.

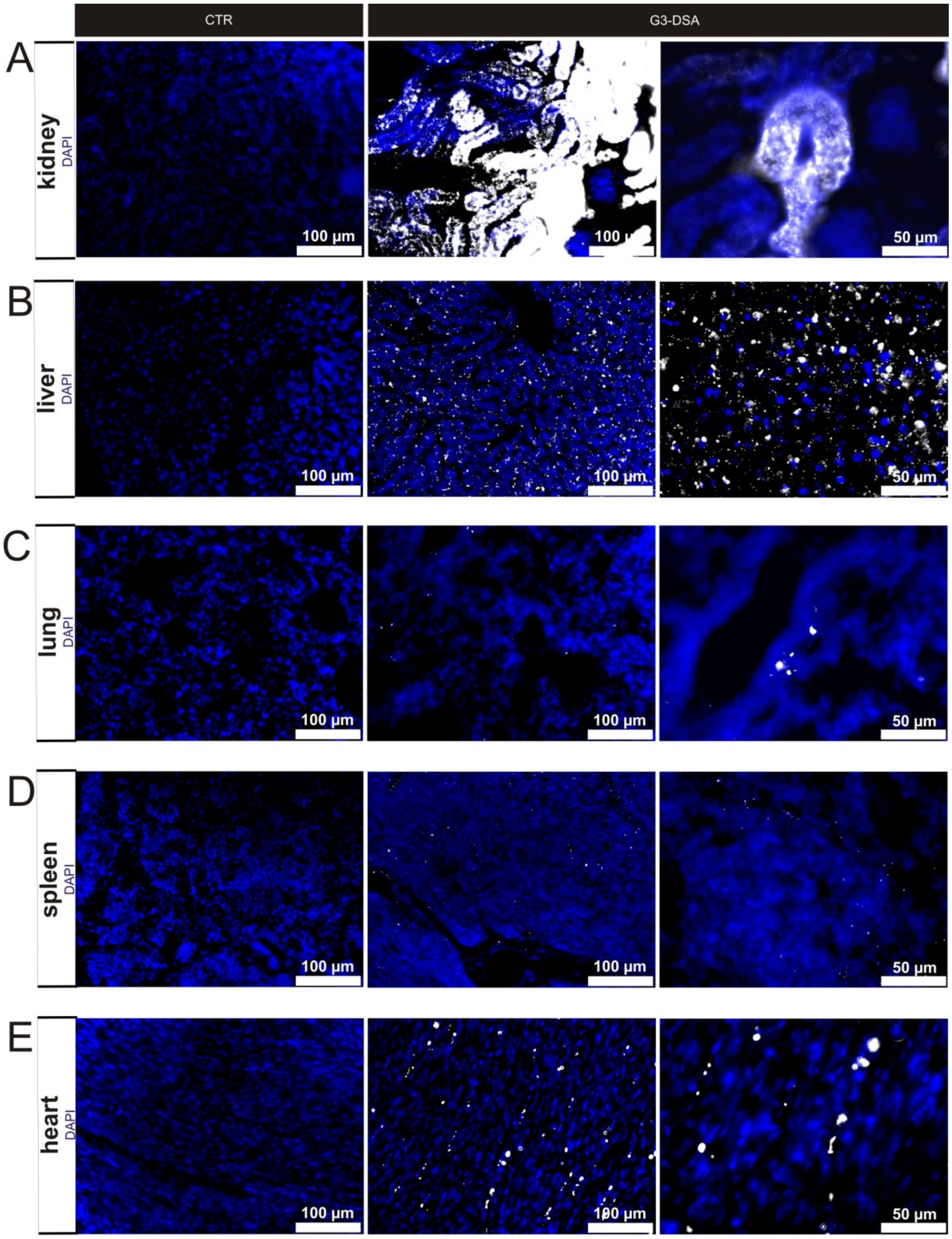

Figure S6. G3-DSA biodistribution in vivo. (A-E) G3-DSA (white) uptake in different organs (kidney **(A)**, liver **(B)**, lung **(C)**, spleen **(D)**, heart **(E)**) 24 h after intravenous injection (450 µg mL^{-1}).

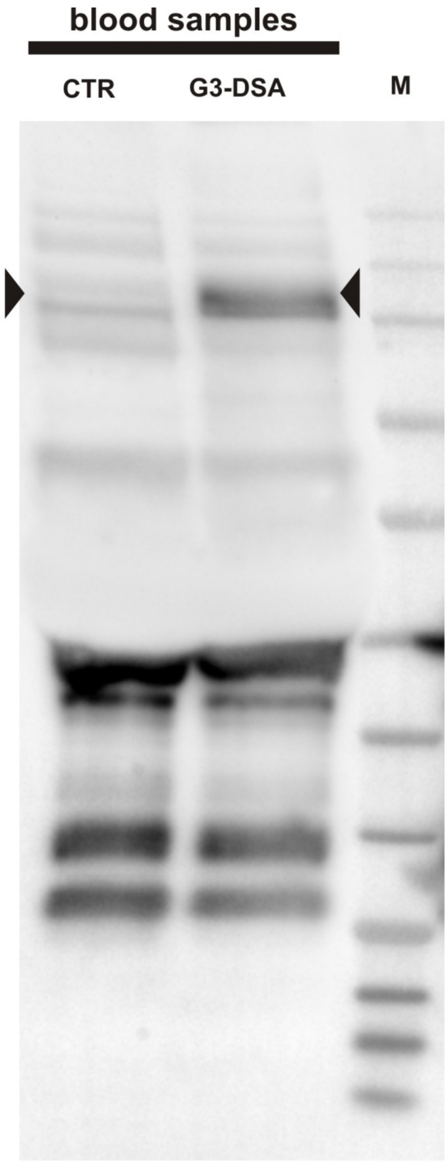

Figure S7. G3-DSA permanence in blood. Streptavidin immunoblotting of blood samples from CTR and G3-DSA treated mice (450 µg mL^{-1}) 24 h post intravenous injection. On PVDF membrane several not specific bands are detectable due to the presence of endogenous antibodies in blood samples. Arrow head: specific band for G3-DSA.

Video 1. DSA endosomal trafficking (CTR early endosome). Representative confocal 3D-view of CTR samples with staining of early endosome antigen (green) for early endosomes. In blue cell nuclei are shown.

Video 2. **DSA endosomal trafficking (CTR late endosome).** Representative confocal 3D-view of CTR samples with staining of lysosomal-associated membrane protein 1 (green) for late endosomes. In blue cell nuclei are shown.

Video 3. **DSA endosomal trafficking (G2-DSA early endosome).** Representative confocal 3D-view of G2-treated samples with staining of early endosome antigen (green) for early endosomes colocalizing with G2-DSA (red). In blue cell nuclei are shown.

Video 4. **DSA endosomal trafficking (G2-DSA late endosome).** Representative confocal 3D-view of G2-treated samples with staining of lysosomal-associated membrane protein 1 (green) for late endosomes colocalizing with G2-DSA (red). In blue cell nuclei are shown.

Video 5. **DSA endosomal trafficking (G2-DSA early endosome).** Representative confocal 3D-view of G2-treated samples with staining of early endosome antigen (green) for early endosomes colocalizing with G3-DSA (red). In blue cell nuclei are shown.

Video 6. **DSA endosomal trafficking (G3-DSA late endosome).** Representative confocal 3D-view of G2-treated samples with staining of lysosomal-associated membrane protein 1 (green) for late endosomes colocalizing with G3-DSA (red). In blue cell nuclei are shown.

Supporting Information

Title: Brain Delivery of Multifunctional Dendrimer Protein Bioconjugates.

Pierpaolo Moscariello, David Y. W. Ng, Malin Jansen, Tanja Weil, Heiko J. Luhmann, Jana Hedrich[*]

Material and Methods

Culturing bEnd.3 Cells: The murine line, bEnd.3, from brain endothelioma (American Type Culture Collection, Manassas, VA, USA) was cultured as recommended by the manufacturer. Cultures were maintained at a humidified atmosphere with 37°C and 5 % CO2. Media consisted of DMEM (Glutamax, gibco by life technology, Darmstadt, Germany) supplemented with 10 % fetal calf serum (Biochrom, S0115) and 2 % penicillin/streptomycin (Invitrogen GmbH, Karlsruhe, Germany). bEnd.3 passages 10–30 were used for experimental assays. Cells were seeded with 80.000 cells per insert (0.3 cm2) or per well in 96-well-plate, 100.000 cells per coverslip, 100.600 cells per ECIS-8-well-chamber.

Primary Murine Astrocytes: Primary astrocyte cultures were generated from six mice (P3). Forebrains were collected and meninges were removed, then the tissue was minced and incubated with 0.05 % trypsin/EDTA for 20 min. Digestion was stopped with 10 % horse serum (HS; S9135, Biochrom) in HBSS -/- (gibco by life technology, Darmstadt, Germany). Cells were dispersed using Pasteur pipettes and filtered by a 40 µm cell strainer. Cells were seeded on poly-ornithine (P4538) coated T75-flasks in culture medium (MEM supplemented with 10 % horse serum and 5 µg mL-1

gentamycin). Cells were subcultured by splitting the cells 1:3. Astrocytes were seeded with 100.000 cells/coverslip, 52.000 cells/well in IBIDI-8-Well-Chamber, 25.000 cell/insert (0.3 cm2).

Primary Murine Neurons: Primary neuronal dissociated cell culture from mice were performed as introduced by Kaech and Banker [1] and optimized by the protocol of Beaudoin and colleagues.[2] Forebrains were collected from mice pups (P0-1) and meninges were removed. Tissue was washed three times with ice cold HBSS -/- and incubated for 20 min in 0.05 % Trypsin/EDTA (gibco by life technology, Darmstadt, Germany). DNase I (Roche, 11284932001) (2000U in 5 mL) was added for 5 min. After 3 washing steps tissue was homogenized in Neuronal Plating Medium (MEM, 10 % horse serum, 0.6 % glucose) and stained with trypan blue (T8154) for cell counting. Primary neurons were seeded with cell numbers of 60.000 cells/well in 96-well-plates or 200.000 cells/coverslip (SDS treated and poly-ornithine coated) in plating medium. After 30 min medium was replaced with Neuronal Maintenance Medium [Neurobasal Medium (gibco by life technology, Darmstadt, Germany) with 2 x 10-3 M Glutamine (gibco by life technology, 25030024) and supplemented with B27 Supplement (gibco, 17504044)]. Cells were fed every 7 days by replacing one-third of the medium with fresh Neuronal Maintenance Medium.

Triple Co-Culture: For the in vitro triple co-culture model primary astrocytes were seeded on day 0 with a cell number of 25.000 cells/insert on the abluminal side of the permeable transwell membrane and 25.000 cells/coverslip. Neurons were grown in Neuronal Maintenance Media on coverslips in separate wells. On day 2-3 bEnd.3 cells (passage 10-30) were seeded with 80.000 cells/insert in the luminal compartment. Co-culture was grown in EBM-2-Basal-Medium (Lonza, Walkersville, USA) supplemented with + 15 % FCS, Glutamine (0.863 mg mL-1) and EBM-2 Single Quot Kit without VEGF growth factor. TEER of co-culture was monitored and the quality benchmark for usage in vitro transport assay was a TEER of at least 27.5 Ωcm2 on day 9-11. On

Day 9-11 of co-culture and DIV 7-9 of neuronal culture inserts were transferred to wells with neurons on the bottom, TEER was measured before and 24 hours after DSA treatment.

Isolation of Porcine Brain Endothelial Cells (PBECs): Porcine brains were collected from butcher Färber in Alzey, Germany and transported on ice in transport buffer (10 % HEPES, 1 % Pen/strep, 1 % Ciprobay 200 Bayer, 1 % Fungizone). Primary brain microvascular endothelial cells were generated as described previously by Freese et al..[3] Two brain hemispheres were incubated 1 h on ice in incubation buffer (10 % HEPES, 1 % Pen/Strep, 1 % Ciprofloxacin Kabi 400 mg/200 mg, 1 % Fungizone). Meninges were peeled off and the gray matter was collected in PBS, minced and centrifuged at 1400 rpm for 10 min at 4°C. Each hemisphere was digested for 30 min at 37°C in DMEM containing 20 mg of Collagenase IV (Worthington; LS004210) and 20 µg of DNase I in a total volume of 3 mL. After washing the tissue was resuspended in a solution of 20 % Percoll (GE Healthcare; 17-0891-01) diluted in PBS and centrifuged at 2600 rpm for 1 h at 4°C. The capillary fragments from both hemispheres were digested for 10 min at 37°C in DMEM containing 2 mg of Collagenase/Dispase (Roche, 10269638061) and 20 µg of DNase I in a total volume of 2 mL and filtered by 100 µm cell strainer. The cell suspension was loaded on a Percoll gradient previously prepared (24 mL Percoll + 13 mL PBS + 2 mL 10x PBS centrifuged at 10000 x g for 2 h at room temperature). The brain endothelial cell fraction was resuspended in EBM-2 medium (Lonza; CC-3156) containing supplements (rhFGF-B, rhEGF, GA-100, Ascorbic Acid, R3-ICF-1, Heparin, Hydrocortisone; Lonza; CC-4114A), 15 % FCS and of Glutamine (0.863 mg mL-1). Cells were seeded in transwell inserts or coverslips precoated with fibronectin (F1141, 5 µg mL-1). Finally, PBECs selection was carried out by puromycin (Enzo; BML-GR312-0050) treatment. At DIV 0 cells were treated with of puromycin (4 µg mL-1, at DIV 1 with 3 µg mL-1 and 2 µg mL-1 at DIV 2).

Determination of Apparent permeability (Papp): was determined using FITC-Dextran 4 (FD4) (46944). FD4 (100 µg mL^{-1}) was applied to the luminal side of the transwell system. After 24 h medium from the abluminal compartment was collected and FD4 fluorescence intensity was measured by Infinite F1000 TECAN plate reader. FD4 concentration was determined in respect to a calibration curve obtained by the measurement of serial dilutions values and the apparent BBB permeability coefficient was calculated according to Artursson et al. 1990,[4] by the following equation 1:

$Papp$ = dQ/dt * 1/A*C_0 [cm s^{-1}].

dQ/dt is the amount of transported FITC-dextran per minute (µg sec^{-1}.), A is the surface area of the filter (0.3 cm^2), C_0 is the initial FITC-dextran concentration (100 µg mL^{-1}).

Immunocytochemistry and Histochemistry: Cells or organs were fixed with PFA 4 %. Probes were blocked and permeabilized with 7 % normal donkey serum (Dianova, Hamburg, Germany)/ 0.3 % (0.8 % for organ slices) Triton (T-8787) in PBS for 2 h at RT. Organ slices were at first incubated with donkey anti-mouse FAB fragment (Dianova, 715-007-003, Hamburg, Germany) in PBS 10 x 10^{-3} M for 2 h at RT. Primary antibody was incubated in 2 % bovine serum albumin (Dianova, 001-000-161, Hamburg, Germany) with 0.05 % azide and 0.1 % (0.3 % for organ slices) Triton/PBS overnight at RT. Secondary antibody and DAPI (32670) were incubated in 2 % bovine serum albumin with 0.05 % azide for 2 h at RT, followed by PBS washing. Organ slices were additionally washed with Tris-HCl (50 x 10^{-3} M) at a pH of 6.5. Cells or slices were finally embedded in Fluoromount (Biozol, Eching, Germany) or CFM-3R (Citifluor Ltd., London, UK).

Table1. Antibodys for immunohistochemistry

**Primary antibody**	**Secondary antibody**

**ZO-1**<br>anti-zonula occludens 1 (rabbit, Zymed via invitrogen, 61-300, 1:100)	Cy2 goat anti-rabbit IgG (Dianova, 111-25-144, 1:10)
**EEA**<br>anti-early endosome antigen-1 (rabbit, Abcam, ab50313, 1:50)	Alexa Fluor 647 donkey anti-rabbit IgG (Jackson, Dianova, 711-605-152, 1:200)
**LAMP1**<br>anti-cluster of differentiation 107a (rat, BD Biosciences, 553792, 1:50)	DyLight 488 anti-rat IgG (Bethyl Lab, Biomol, 1:200)
**VAMP3**<br>anti-vesicle-associated membrane protein 3 (rabbit, Synaptic System, 1:250)	Cy2 donkey anti-rabbit IgG (Jackson, Dianova, 711-225-152, 1:200)
**TfR**<br>anti-transferrin receptor (mouse, Zymed via Invitrogen, 1:500)	Dylight 488 donkey anti-mouse IgG (Bethyl Lab, Biomol, A90-337D2, 1:200)
**LC3**<br>anti-microtubule-associated protein 1A/1B-light chain 3 (mouse, Nanotools, 0260S/LC3-2G6, 1:200)	Dylight 488 donkey anti-mouse IgG (Bethyl Lab, Biomol, A90-337D2, 1:200)
**GFAP**<br>anti-glial fibrillary acidic protein (mouse, Synaptic Systems, 173011)	Dylight 488 donkey anti-mouse IgG (Bethyl Lab, Biomol, A90-337D2, 1:200)
**NeuN**<br>anti-neuronal nuclear antigen (rabbit, Merck Millipore, ABN78)	Dylight 488 donkey anti-rabbit IgG (Jackson, Dianova, 711-485-152, 1:200)

**CD105** anti-endoglin (rat, Beckman Coulter, 732334)	DyLight 488 anti-rat IgG (Bethyl Lab, Biomol, 1:200)

Protein Concentration: Culture medium after transport assay from abluminal compartment was centrifuged at 14000 x g using Amicon Ultra 10K centrifugal filter devices (UFC501024; Millipore) to concentrate the protein in a volume of 20 μL according to the provided protocol.

C*ell Lysate:* bEnd.3 cells were scraped off in culture medium and the suspension was centrifuged for 5 min at 4°C at 200 x g. The pellet was washed with PBS and centrifuged for 5 min at 4°C at 400 x g, dissolved in 1 volume of lysis buffer (50 x 10^{-3} M Tris; 150 x 10^{-3} M NaCl; 1 x 10^{-3} M EDTA; 1 % Triton-X-100) supplemented with protease inhibitor (Roche) and incubated 45 min on rotation wheel at 4°C. The cell lysate was obtained collecting the supernatant after centrifugation for 5 min at 4°C at 13000 x g. The pellet was also dissolved in 1 volume of lysis buffer to obtain the cell pellet probe. Cell lysate and cell pellet probes were stored at -20°C.

Western Blot Analysis: Samples were separated by electrophoresis on a SDS-Polyacrylamide gel and transferred onto a polyvinylidene difluoride membrane. The membrane was blocked with non-fat 4 % milk in TBST (500 x 10^{-3} M Tris base; 1.5 M NaCl; pH 7.2; 0.1 % Tween 20). Antibodies against streptavidin (ab10020; 1:500; Abcam), LC3 (mab LC3-2G6; 1:200; Nanotools) or GAPDH (A300-641A, 1:5000, Bethyl Laboratories) diluted in non-fat 4 % milk in TBST were applied. Finally, the protein of interest was detected by HRP-conjugated anti-mouse antibody (115-035-003; 1:5000; Dianova) or HRP-conjugated anti-rabbit antibody (111-035-144; 1:10000; Dianova) and

visualized by ECL Western Blotting substrate (Merck Millipore) according to the provided protocol.

Analysis of Autophagy Induction by LC3-II Immunoblotting: bEnd.3 cells seeded in 24 well/plate were treated for 24 h with DSA (45 µg mL^{-1}). In the last 4 h of the 24 h treatment period, cells were incubated with BAF (400 x 10^{-9} M) to inhibit autophagosome/lysosome fusion. Cell lysates were then used for WB detection of endogenous LC3-II.

Analysis of DSA Short-Term Lysosomal Degradation: bEnd.3 cells seeded in 24 well/plate were treated for 24 h with of DSA (45 µg mL^{-1}). A concentration of BAF (400 x 10^{-9} M) was applied for 24 h in co-treatment with DSA or in the last 4 h of the DSA treatment to inhibit lysosomal entrapment and degradation of DSA. Cell lysates were then used for WB detection of streptavidin core.

Supplementary Figures:

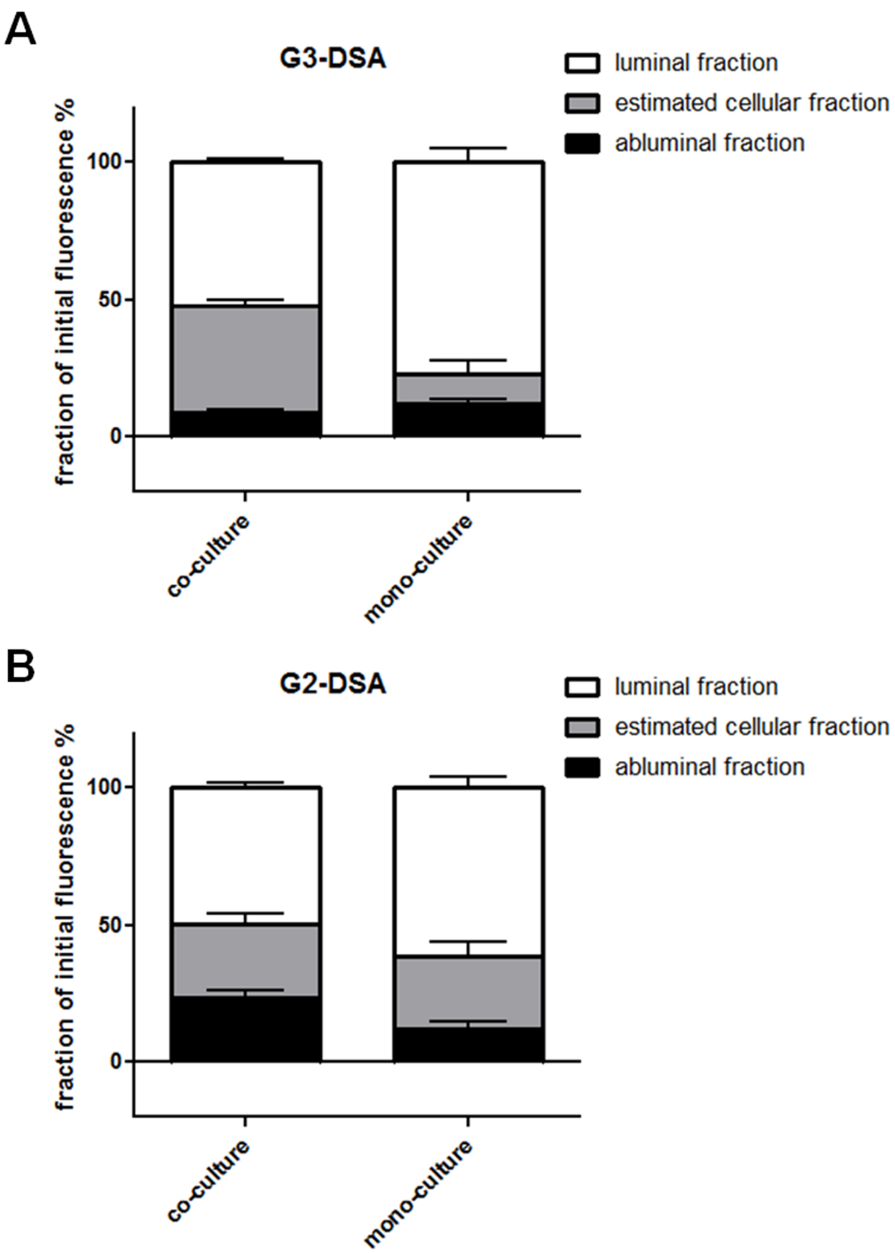

Figure S1. DSA fractions of BBB in vitro models. Three different fractions of fluorescence in the transwell model are blotted. After 24 h of G3-DSA **(A)** or G2-DSA **(B)** application in luminal compartment. Estimated cellular fraction = DSA initial fluorescence - DSA abluminal fluorescence - DSA luminal fluorescence. Co-culture: n = 8; mono-culture: n = 6.

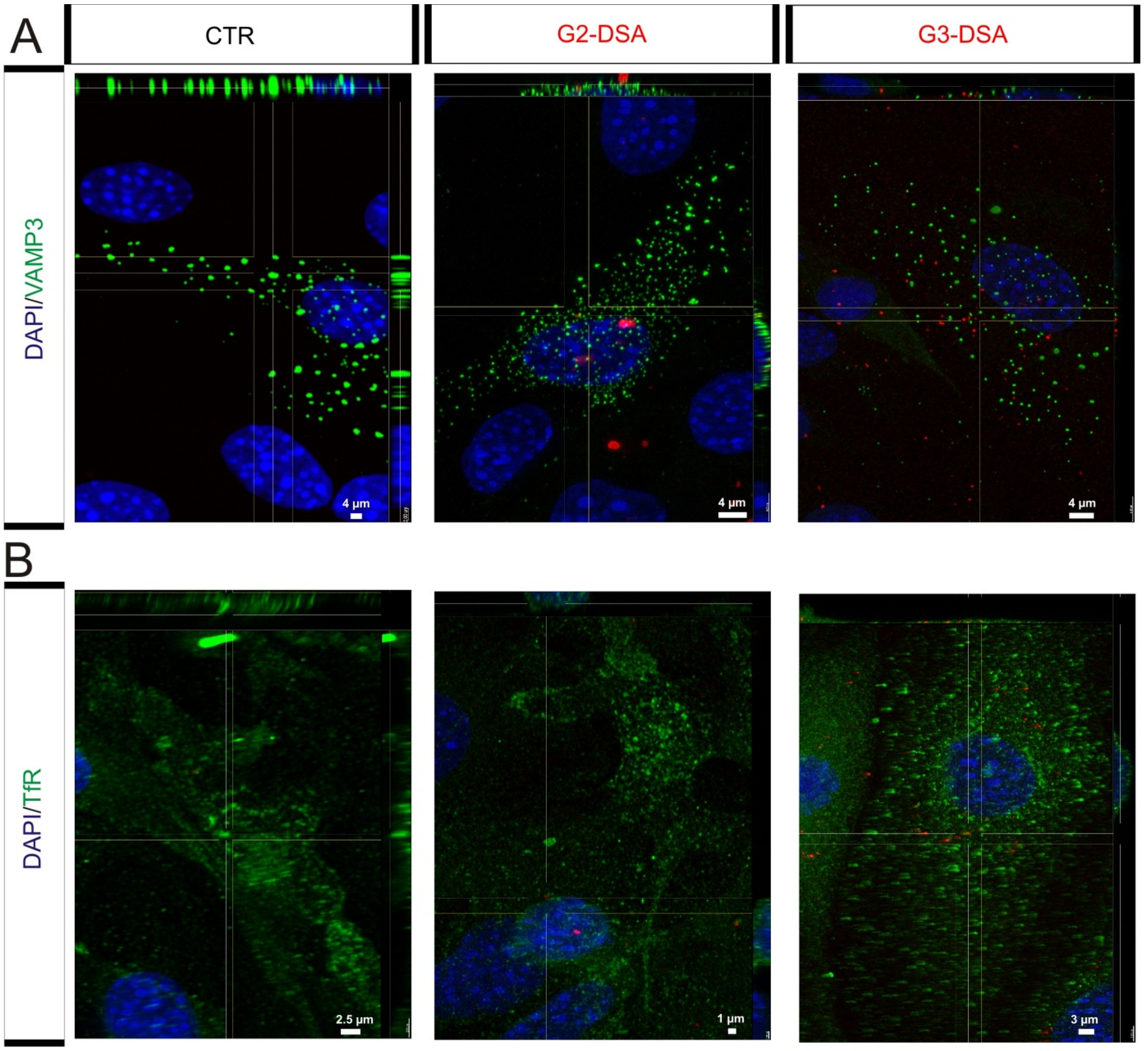

Figure S2. DSA intracellular trafficking. Representative confocal tridimensional orthogonal view for colocalization analysis of DSA-positive vesicles (red) and vesicle associated membrane protein 3 (VAMP3; green) **(A)** for recycling endosomes or transferrin receptor (TfR; green) **(B)**.

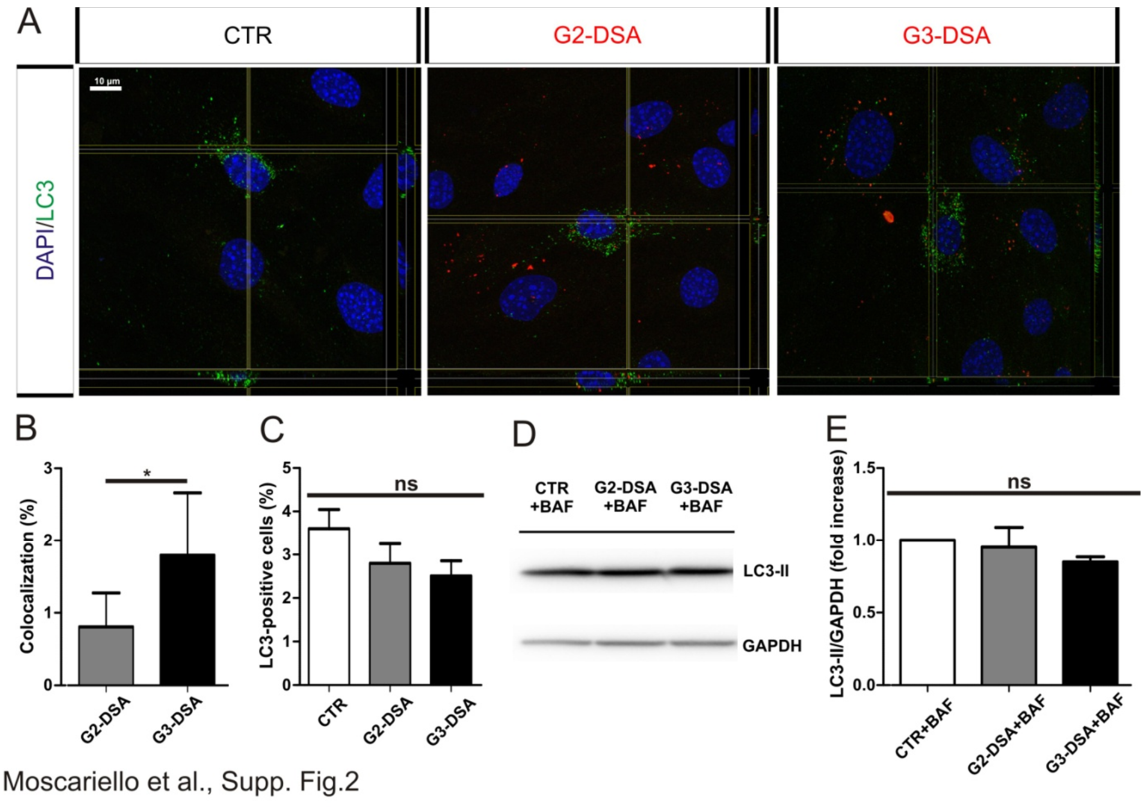

Figure S3. Effect of DSA on autophagy. (A) Representative confocal tridimensional orthogonal view for colocalization analysis of DSA-positive vesicles (red) and microtubule-associated protein 1A/1B-light chain 3 (LC3; green). **(B)** Quantification of colocalization for DSA with autophagosomes. Data are expressed in percentage of DSA-positive-vesicles partially colocalizing for at least 40 % with autophagosome-vesicles. G2-DSA n = 20 ROIs, G3-DSA n = 17 ROIs from 3 cultures; Mann-Whitney U test, $*p < 0.05$. **(C)** Percentage of LC3-positive cells in CTR or DSA treated bEnd.3 cells. Total number of cells in each analyzed ROI represents 100%. n = 30 ROIs from 3 cultures; one-way ANOVA; ns = not significant. **(D, E)** Western blot of lysates **(D)** and bands densitometric quantification **(E)** of LC3-II levels in CTR and DSA-treated cells. Bafilomycin (BAF) was used as inhibitor of autophagosome-lysosome fusion.n = 3 cell lysates from 3 cultures; one-way ANOVA; ns = not significant.

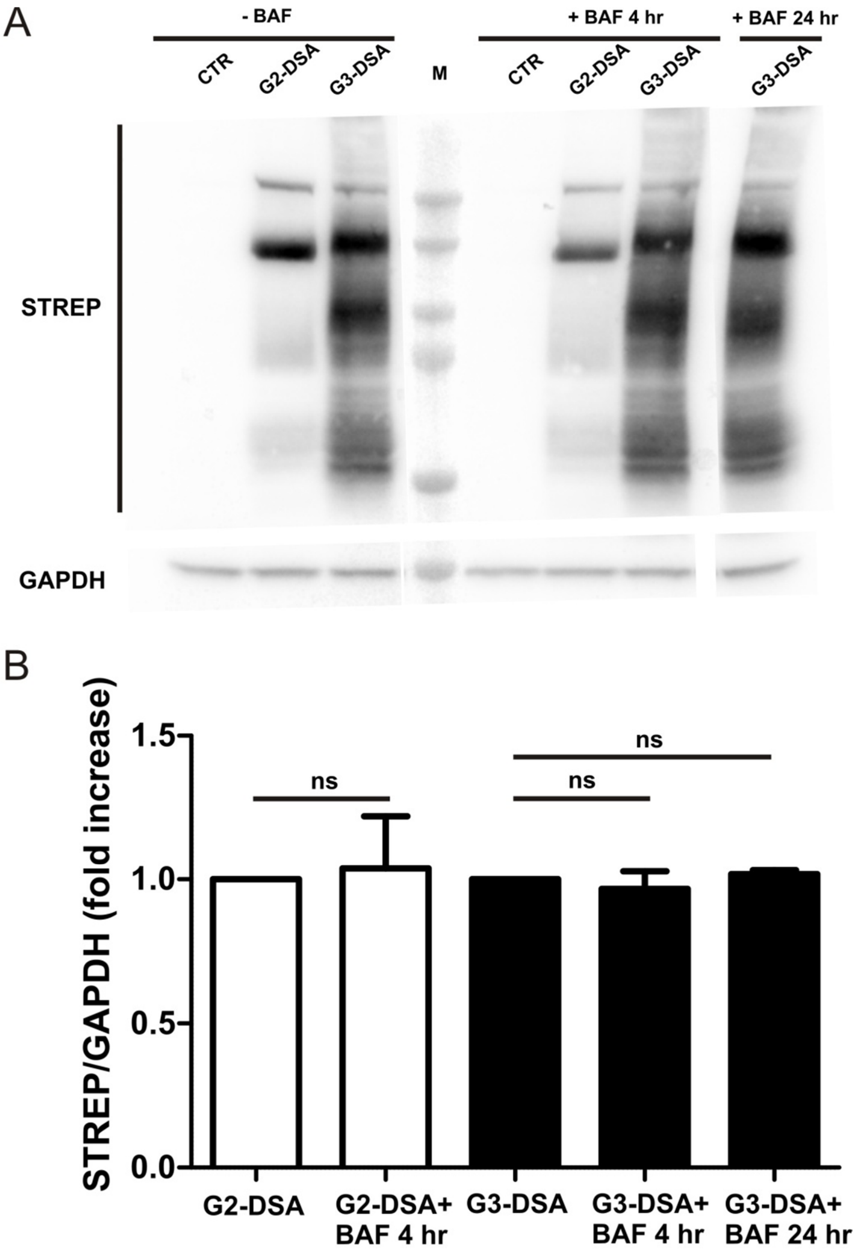

Figure S4. DSA lysosomal degradation. (A) Streptavidin (STREP) immunoblotting of lysate from CTR and DSA treated bEnd.3 cells. Bafilomycin (BAF) treatment for 4 h or 24 h was used to induce lysosomal escape of DSA. **(B)** Densitometric quantification of STREP levels for DSA-treated cells with or without BAF treatment. STREP levels of not BAF-treated samples were set to 1 and each correspondent BAF-treatment expressed as relative value; n = 3 cell lysates from 3 cultures; one-way ANOVA; ns = not significant.

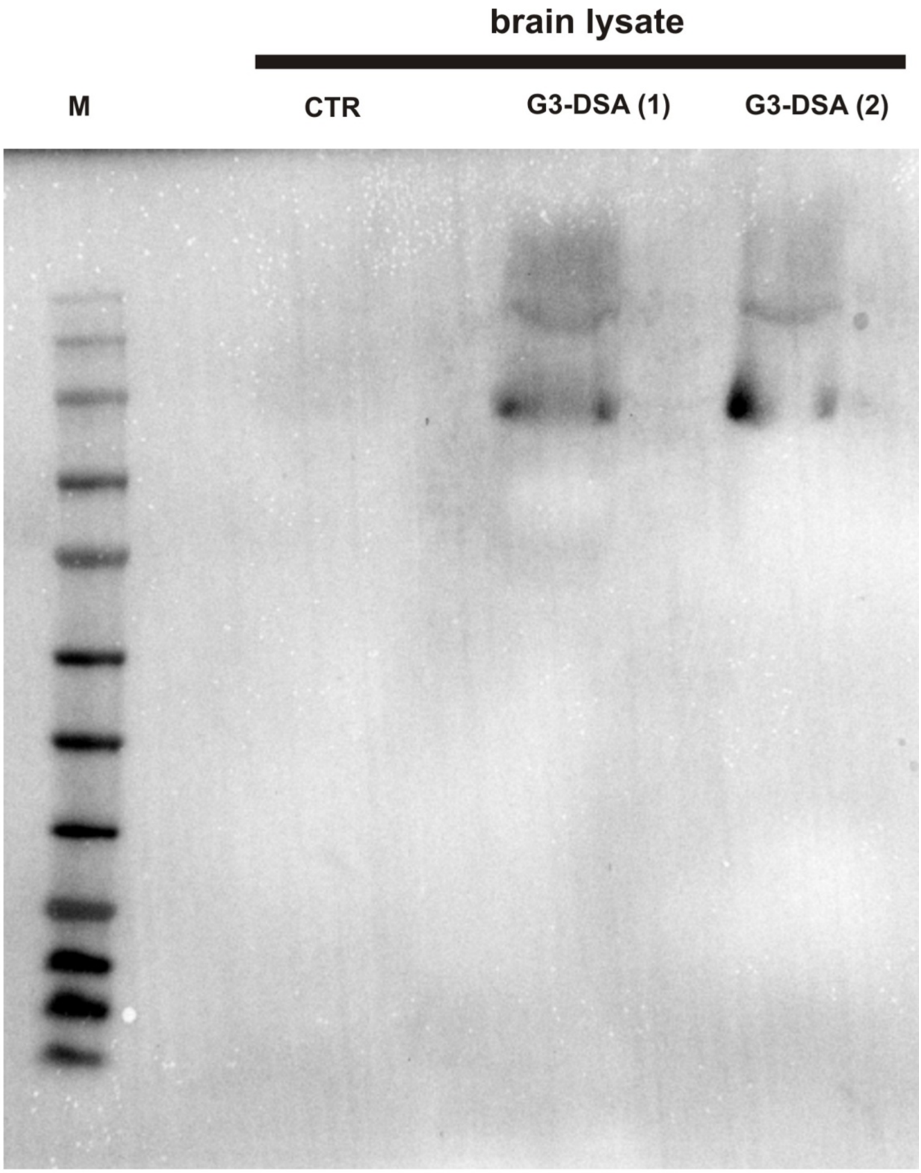

Figure S6. G3-DSA brain uptake in vivo. Streptavidin immunoblotting of brain lysate from CTR and 2 x G3-DSA treated mice (450 µg mL^{-1}) 24 h post intravenous injection.

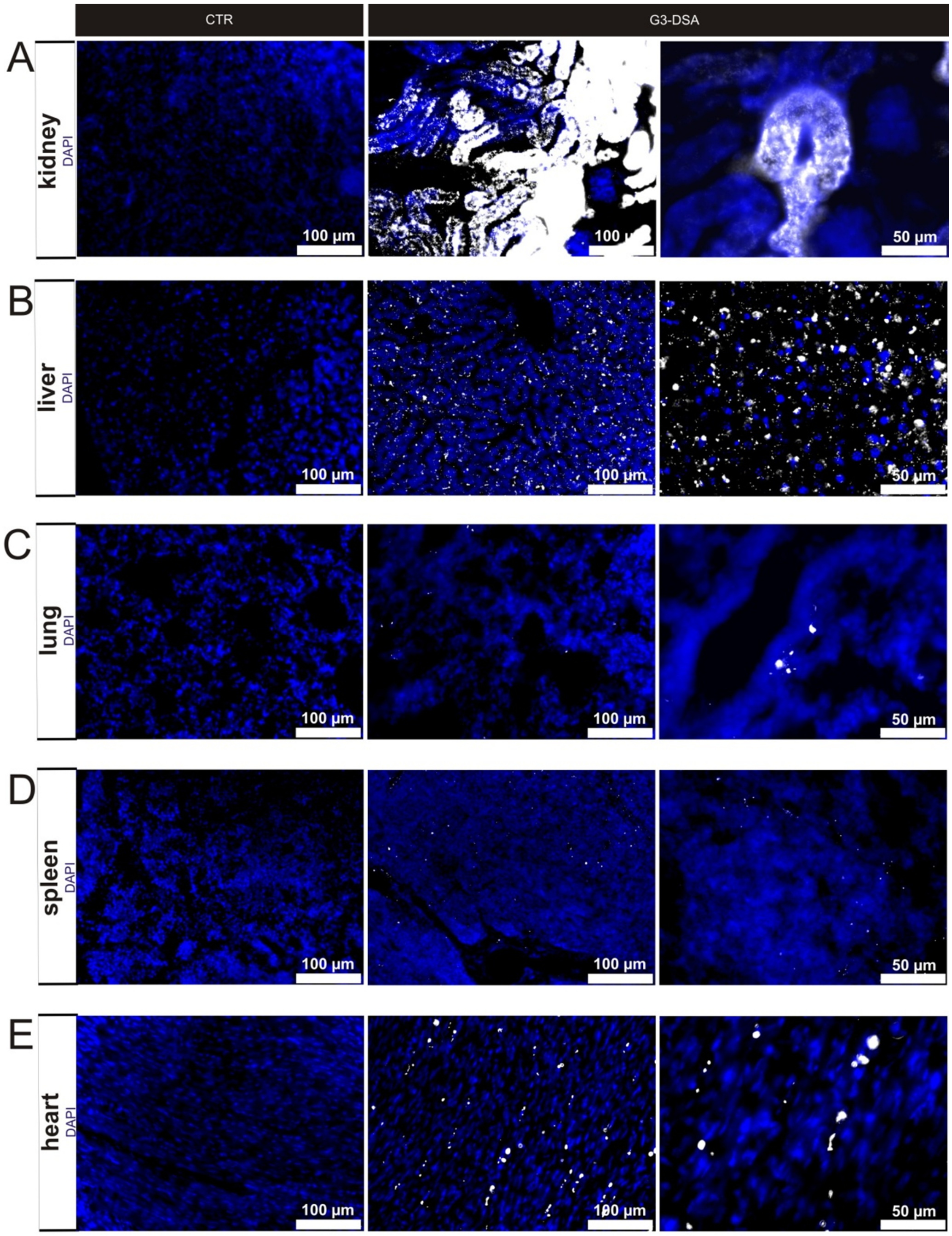

Figure S7. G3-DSA biodistribution in vivo. (A-E) G3-DSA (white) uptake in different organs (kidney **(A)**, liver **(B)**, lung **(C)**, spleen **(D)**, heart **(E)**) 24 h after intravenous injection (450 µg mL^{-1}).

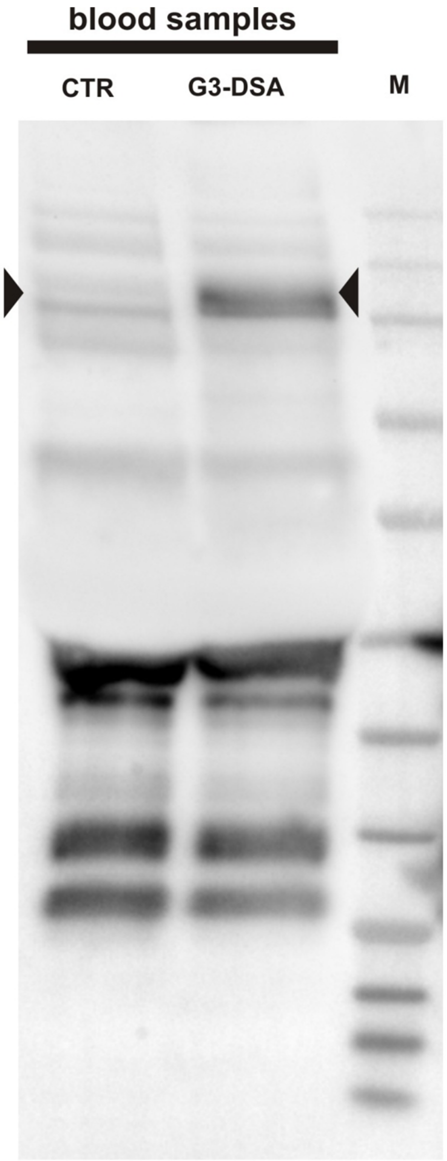

Figure S8. G3-DSA permanence in blood. Streptavidin immunoblotting of blood samples from CTR and G3-DSA treated mice (450 µg mL^{-1}) 24 h post intravenous injection. On PVDF membrane several not specific bands are detectable due to the presence of endogenous antibodies in blood samples. Arrow head: specific band for G3-DSA.

Video 1. DSA endosomal trafficking (CTR early endosome). Representative confocal 3D-view of CTR samples with staining of early endosome antigen (green) for early endosomes. In blue cell nuclei are shown.

Video 2. **DSA endosomal trafficking (CTR late endosome).** Representative confocal 3D-view of CTR samples with staining of lysosomal-associated membrane protein 1 (green) for late endosomes. In blue cell nuclei are shown.

Video 3. **DSA endosomal trafficking (G2-DSA early endosome).** Representative confocal 3D-view of G2-treated samples with staining of early endosome antigen (green) for early endosomes colocalizing with G2-DSA (red). In blue cell nuclei are shown.

Video 4. **DSA endosomal trafficking (G2-DSA late endosome).** Representative confocal 3D-view of G2-treated samples with staining of lysosomal-associated membrane protein 1 (green) for late endosomes colocalizing with G2-DSA (red). In blue cell nuclei are shown.

Video 5. **DSA endosomal trafficking (G2-DSA early endosome).** Representative confocal 3D-view of G2-treated samples with staining of early endosome antigen (green) for early endosomes colocalizing with G3-DSA (red). In blue cell nuclei are shown.

Video 6. **DSA endosomal trafficking (G3-DSA late endosome).** Representative confocal 3D-view of G2-treated samples with staining of lysosomal-associated membrane protein 1 (green) for late endosomes colocalizing with G3-DSA (red). In blue cell nuclei are shown.

References

[1] S. Kaech, G. Banker, *Nat Protoc* **2006**, 1, 2406.

[2] G. M. Beaudoin, S. H. Lee, D. Singh, Y. Yuan, Y. G. Ng, L. F. Reichardt, J. Arikkath, *Nat Protoc* **2012**, 7, 1741.

[3] C. Freese, S. Reinhardt, G. Hefner, R. E. Unger, C. J. Kirkpatrick, K. Endres, *PLoS One* **2014**, 9, e91003.

[4] P. Artursson, *J Pharm Sci* **1990**, 79, 476.
